# Efficacy and safety of Xiangju capsules for allergic rhinitis: a systematic review and meta-analysis of randomized controlled trials

**DOI:** 10.3389/fphar.2026.1788708

**Published:** 2026-05-28

**Authors:** Min Chen, Ya-Wen Lu, Jun Shi, Ping-Min Ni, Xiao-Ning Chen

**Affiliations:** 1 Jiangsu Province Hospital of Chinese Medicine, Affiliated Hospital of Nanjing University of Chinese Medicine, Nanjing, Jiangsu, China; 2 Nanjing University of Chinese Medicine, Nanjing, Jiangsu, China

**Keywords:** allergic rhinitis, meta-analysis, randomized controlled trials, systematic review, traditional Chinese medicine, xiangju capsules

## Abstract

**Background:**

Allergic rhinitis (AR) is a common chronic inflammatory disease, which seriously affects patients’ quality of life. Xiangju (XJ) capsules are often used as an adjunctive drug for AR treatment in clinical practice, but its efficacy and safety have not been systematically evaluated.

**Methods:**

This study systematically searched eight Chinese and English databases for data from inception to 30 November 2025. Randomized controlled trials (RCTs) evaluating XJ capsules alone or in combination with conventional treatment for AR were included. Subgroup analyses and sensitivity analyses were used to test outcome stability. Funnel plots, Egger’s regression test and Begg’s rank correlation test were used to assess publication bias. When publication bias was detected, its potential influence was further assessed using the trim-and-fill method. The methodological quality of the included studies was evaluated using the Cochrane Risk of Bias tool, and the quality of evidence was graded using the Grading of Recommendations Assessment, Development and Evaluation (GRADE) framework.

**Results:**

A total of 14 RCTs involving 1,968 patients were included. Meta-analysis showed that XJ capsules were associated with a higher reported overall effective rate (RR = 1.12, 95% CI: 1.08 to 1.16), lower runny nose scores (SMD = −1.81, 95% CI: −3.36 to −0.27) and lower sneezing scores (SMD = −1.14, 95% CI: −1.86 to −0.41), lower IL-4 levels (SMD = −1.55, 95% CI: −1.93 to −1.16) and lower IgE levels (SMD = −1.55, 95% CI: −2.92 to −0.18), and higher IL-12 levels (SMD = 1.76, 95% CI: 0.79 to 2.73). No significant difference was observed in adverse event incidence between groups (RR = 0.72, 95% CI: 0.43 to 1.20).

**Conclusion:**

XJ capsules may offer additional benefits for AR symptoms and immunological markers, and were generally well tolerated in the included trials. However, given the limited certainty of the evidence, further high-quality studies are needed to confirm these findings.

**Systematic Review Registration:**

https://www.crd.york.ac.uk/PROSPERO/view/CRD420261279480, identifier CRD420261279480.

## Introduction

Allergic rhinitis (AR) is a prevalent chronic inflammatory disorder of the upper airways manifested by itching, sneezing, runny nose, and nasal congestion, and commonly co-occurs with asthma and other atopic disorders (Klimek et al., 2024). Current international consensus documents indicate that, in addition to localized nasal symptoms, AR significantly affects patients’ quality of life, sleep status, as well as daily functioning, imposing a significant socioeconomic burden by reducing learning and work productivity (Wise et al., 2023; Meltzer, 2016; Jones et al., 2025; Vieira et al., 2024).

Current authoritative guidelines for AR generally recommend nasal glucocorticoids, nasal antihistamines, and oral antihistamines as first-line treatments, and consider combination regimens or allergen-specific immunotherapy in patients with poor outcomes (Wise et al., 2023; Sousa-Pinto et al., 2025; Dykewicz et al., 2020). However, although the above treatment strategies have been proven effective at the evidence-based level, real-world studies have shown that a significant proportion of AR patients still fail to achieve satisfactory symptom control (Noreña-Pe et al., 2025; Baharudin et al., 2025). In addition, some patients have concerns about the safety of long-term medication or experience symptomatic relapse after discontinuation, prompting clinicians to explore complementary or alternative strategies to conventional drug therapy (Baharudin et al., 2025). The guideline update also emphasizes that AR is highly heterogeneous in terms of clinical presentation and treatment response, and that factors such as patient preference, sensory experience of the medication, financial burden, and long-term adherence are becoming important determinants of treatment outcomes (Sousa-Pinto et al., 2025; Noreña-Pe et al., 2025).

In recent years, there has been increasing evidence suggesting that traditional Chinese medicine (TCM) treatments, especially as adjuncts to conventional treatments, may play a positive role in improving symptom control and modulating immune responses associated with allergic inflammation. A recent meta-analysis of pediatric AR patients showed that TCM treatment significantly alleviated clinical nasal symptoms and was accompanied by favorable changes in IgE and multiple cytokine levels (Chen et al., 2023). In addition, relevant studies have pointed to the clear efficacy of Chinese botanical drugs in the treatment of AR, which are effective in improving nasal symptoms in seasonal and perennial AR (11). Xiangju (XJ) capsules are a widely used proprietary Chinese medicine in Chinese otorhinolaryngology clinical practice, often used as adjunctive therapy for patients with AR. Although several randomized controlled trials (RCTs) have evaluated the clinical efficacy of XJ capsules in the treatment of AR, its overall efficacy and safety profile still lacks systematic and focused integration of evidence. In addition, there are large differences in the study populations, combination regimens and treatment duration in the existing studies, which may limit its clinical dissemination value. From a pharmacological perspective, existing pharmacological evidence provides a basis for evaluating immune-related outcomes in studies of Xiangju Capsules. A network pharmacology study on Xiangju formulations suggests that its potential anti-allergic effects may involve multiple inflammation- and immune-related targets and pathways associated with allergic rhinitis, including cytokine-mediated inflammatory responses, IgE-related signal transduction, and Th2-related immune regulation (Hu et al., 2019). Meanwhile, experimental studies on Magnoliae Flos, one of the botanical drugs in this formulation, have shown that it can inhibit mast cell-dependent immediate-type allergic reactions and suppress the release of histamine by mast cells (Shen et al., 2008; Kim et al., 1999). AR is closely associated with Th1/Th2 immune imbalance, in which IL-4 promotes IgE-mediated allergic reactions, while IL-12 exerts a negative regulatory effect on Th2-biased inflammation (Liang and Gu, 2021). Together, these findings provide pharmacological support for the potential anti-allergic and immunomodulatory effects of XJ capsules in AR.

Based on the above research gaps, we comprehensively assessed the efficacy and safety of XJ capsules in the treatment of AR by conducting a systematic review and meta-analysis of RCTs. This study aimed to provide clinicians and researchers with a comprehensive evidence synthesis to clarify the potential role of XJ capsules in the adjunctive management of AR.

## Methods

This study followed the Preferred Reporting Items for Systematic Reviews and Meta-Analyses (PRISMA) framework in its conduct and reporting (Page et al., 2021). The protocol was prospectively registered with the International Prospective Register of Systematic Reviews (PROSPERO) under the number CRD420261279480. The PRISMA 2020 checklist is provided in Supplementary Table S1.

### Data sources and search strategy

A comprehensive literature search was performed across the following electronic databases: China National Knowledge Infrastructure (CNKI), Wanfang Data, VIP Database, China Biology Medicine (CBM), PubMed, Embase, the Cochrane Library, and Web of Science. All databases were searched from their respective inception dates to 30 November 2025. Only studies published in Chinese or English were included. The types of literature searched included journal articles, postgraduate dissertations, conference papers and other related literature. The primary search terms included “Xiangju capsule,” “Xiangju,” “XJ capsule,” along with their relevant synonyms. The complete search strategies for all databases are provided in Supplementary Table S2.

### Description of xiangju capsules

XJ capsules are a commercial proprietary Chinese medicinal product. According to the manufacturer’s official product information, XJ capsules contain Huaxiangshu fruit infructescence with seeds removed, *Platycarya strobilacea* Siebold and Zucc. [Juglandaceae]; *Prunella vulgaris* L. [Lamiaceae; Prunellae Spica]; *Chrysanthemum indicum* L. [Asteraceae; Chrysanthemi Indici Flos]; *Astragalus mongholicus* Bunge [Fabaceae; Astragali Radix]; *Magnolia biondii* Pamp. [Magnoliaceae; Magnoliae Flos]; *Saposhnikovia divaricata* (Turcz. ex Ledeb.) Schischk. [Apiaceae; Saposhnikoviae Radix]; *Angelica dahurica* (Hoffm.) Benth. and Hook.f. ex Franch. and Sav. [Apiaceae; Angelicae Dahuricae Radix]; *Glycyrrhiza uralensis* Fisch. [Fabaceae; Glycyrrhizae Radix et Rhizoma]; and *Ligusticum chuanxiong* Hort. [Apiaceae; Chuanxiong Rhizoma]. Maize starch and magnesium stearate are listed as excipients. The product is manufactured by Shandong Buchang Pharmaceutical Co., Ltd., with a specification of 0.3 g per capsule (equivalent to 3.186 g crude drug per capsule), under Chinese drug approval number Z19991040 and national drug standard WS3-161(Z-151)-2001(Z). In the included RCTs, XJ capsules were used as an adjunctive intervention for allergic rhinitis. However, as this study synthesized published clinical reports, detailed information on batch-to-batch quality attributes, certificate of analysis, and product-specific phytochemical fingerprinting was not consistently reported in the original trials and could not be comprehensively assessed. The detailed composition of XJ capsules, together with the source and availability of formulation information in each included study, is summarized in Supplementary Table S3.

### Inclusion criteria

The inclusion criteria were as follows (Klimek et al., 2024): Study population: patients diagnosed with AR, regardless of age, sex, disease duration, or disease severity (Wise et al., 2023); Intervention: the experimental group was treated with XJ capsules, either alone or in combination with conventional biomedical treatment (Meltzer, 2016); Control: the control group received the same conventional biomedical treatment without XJ capsules, including antihistamines, nasal glucocorticoids, or other standard AR treatment (Jones et al., 2025); Outcome: studies reported at least one of the following outcome measures: overall effective rate, sneezing symptom score, runny nose symptom score, nasal congestion symptom score, interleukin-4 (IL-4), interleukin-12 (IL-12), immunoglobulin E (IgE), or adverse events (Vieira et al., 2024); Study design: only RCTs published in Chinese or English were included.

The overall effective rate referred to the proportion of patients demonstrating a clinical response to treatment, including marked improvement or complete relief of the main symptoms of AR (nasal congestion, runny nose, sneezing, and itchy nose). Across the included RCTs, this outcome was assessed using largely consistent criteria based on symptom improvement. No improvement or worsening of symptoms was judged to be ineffective.

Symptom outcomes were assessed using the symptom severity scoring systems reported in the original studies. Three studies employed the nasal symptom and sign scoring system described in the Principles and Recommendations for the Diagnosis and Treatment of Allergic Rhinitis (Lanzhou, 2004), which classifies primary nasal symptoms into grades 0 to 3, with higher scores indicating more severe symptoms. One study used a TCM syndrome scoring system based on the Standards for Diagnosis and Therapeutic Effect of TCM Diseases, in which primary nasal symptoms were classified into grades 0 to 3, with higher scores indicating more severe symptoms.

### Exclusion criteria

The exclusion criteria were as follows (Klimek et al., 2024): duplicate publications or studies based on overlapping data (Wise et al., 2023); non-RCTs, including observational studies, case reports, reviews, animal experiments, and *in vitro* studies (Meltzer, 2016); studies in which the diagnosis of AR was not clearly stated (Jones et al., 2025); studies in which the experimental group used additional adjunctive interventions not used in the control group, such as other botanical drug interventions, acupuncture, moxibustion, or other non-pharmacological therapies, making it difficult to independently evaluate the effect of XJ capsules (Vieira et al., 2024); studies in which the control group did not receive comparable conventional biomedical treatment (Sousa-Pinto et al., 2025); studies with unavailable full text, insufficient outcome data, or data that could not be extracted for meta-analysis.

### Study selection and data extraction

First, duplicates were removed from all retrieved records (M.C.). Subsequently, an initial screening of titles and abstracts was performed to exclude clearly irrelevant studies (M.C. and Y.-W.L.). Full texts of potentially eligible studies were then assessed in detail according to the predefined inclusion and exclusion criteria (M.C. and Y.-W.L.). Data were independently extracted, including first author, year of publication, sample size, participant characteristics, interventions and controls, treatment duration, outcome indicators, and methodological characteristics (M.C. and Y.-W.L.). Any disagreements were resolved by discussion or, if necessary, adjudicated by a third researcher (J.S.).

### Risk of bias assessment

Risk of bias in the included studies was evaluated using the Cochrane Risk of Bias tool in accordance with the guidance provided by the Cochrane Handbook (Higgins et al., 2011). Each study was assessed independently by two researchers (M.C. and Y.-W.L.) on seven aspects (Klimek et al., 2024): the method of generating the randomized sequence (Wise et al., 2023); whether appropriate allocation concealment was applied (Meltzer, 2016); whether the study subjects and investigators were blinded (Jones et al., 2025); whether blinding of outcome assessors was implemented (Vieira et al., 2024); whether outcome data were complete and adequately reported (Sousa-Pinto et al., 2025); whether selective reporting bias was present; and (Dykewicz et al., 2020) whether there were other possible potential sources of bias. The risk of bias for each dimension was determined as “low risk,” “high risk,” or “unclear risk”. In the event of disagreement during the assessment, issues were resolved by discussion between the two reviewers (M.C. and Y.-W.L.). If no agreement could be reached, a third researcher (J.S.) was involved in the adjudication process.

### Quality of evidence

Evidence certainty across outcomes was evaluated according to the Grading of Recommendations Assessment, Development, and Evaluation (GRADE) framework (Liu, 2022). Five domains were considered, including risk of bias, inconsistency, indirectness, imprecision, and publication bias. Based on these criteria, the quality of evidence was rated as high, moderate, low, or very low.

### Statistical analysis

Binary outcomes were pooled as risk ratios (RRs) with 95% confidence intervals (CIs). For continuous variables, standardized mean differences (SMDs) and corresponding 95% CIs were calculated because symptom scoring methods and laboratory measurement units varied across the included studies. Statistical heterogeneity among studies was quantified using the *I* ^*2*^ statistic, and random-effects models were used when heterogeneity was substantial (*I* ^*2*^ > 50%). Otherwise, a fixed-effects model was applied. To examine the stability of the pooled estimates, sensitivity analyses were performed by leave-one-out method. Publication bias was explored for outcomes with ten or more included studies by the funnel plots, complemented by Egger’s regression and Begg’s rank correlation tests. If publication bias was detected, its potential effect on the pooled outcomes was assessed using the trim-and-fill method. Data analyses were carried out with R software (version 4.3.2) using the meta package and Review Manager (RevMan) version 5.3.

## Results

### Study selection

**FIGURE 1:**
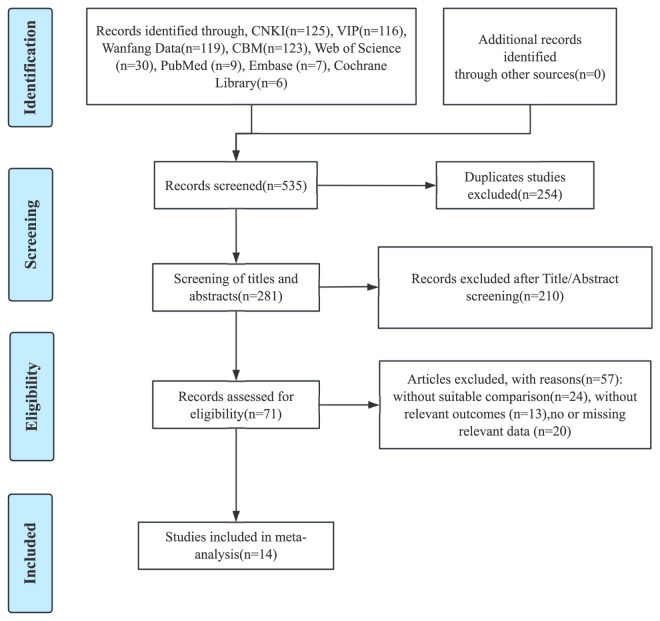
PRISMA flow diagram of study selection.

The initial database search obtained 535 documents from sources including CNKI (n = 125), VIP (n = 116), Wanfang Database (n = 119), CBM (n = 123), Web of Science (n = 30), PubMed (n = 9), Embase (n = 7), and Cochrane Library (n = 6). No additional literature was obtained from other sources. After removing 254 duplicate records, 281 articles proceeded to the title and abstract screening phase. Two hundred and ten studies were excluded for irrelevance to the research question, and 71 articles proceeded to full-text assessment. From these studies, 57 were excluded for the reasons described below: lack of an appropriate control group (n = 24), failure to report relevant outcome measures (n = 13), or incomplete or missing outcome data (n = 20). A total of 14 RCTs met the eligibility criteria and were incorporated into the final meta-analysis. Figure 1 depicts the detailed screening process.

### Characteristics of the included studies

This review included 14 RCTs (Fu and Qian, 2023; Wang et al., 2022; Zhang, 2020; Yang and Liu, 2019; He and Ma, 2019; Yang, 2018; Wang, 2018; Zhang, 2016; Zhang et al., 2016; Liang, 2016; Sun and Yang, 2015; Cao, 2014; Shi and Zhao, 2012; Shi et al., 2010) published between 2010 and 2023, involving a total of 1,968 patients with AR (Table 1). Sample sizes ranged from 60 to 240 participants. Study populations comprised children (4 studies), adults (9 studies), and one mixed-age study, with treatment durations varying from 10 days to 6 weeks. All studies compared the efficacy of XJ capsules combined with conventional therapy *versus* conventional therapy alone. Concomitant medications primarily included oral antihistamines, intranasal corticosteroids, azelastine, montelukast, or oxymetazoline. XJ capsules were administered at 2–4 capsules per dose, 2–3 times daily. Clinical outcomes assessed included overall effective rate, symptom scores (rhinorrhea, sneezing, nasal congestion, nasal itching), serum IgE, IL-4, IL-12, and adverse events. The availability and sources of formulation details are summarized in Supplementary Table S3.

**TABLE 1:** Characteristics of included studies.

Study	Sample size		Age (Year)		Male/Female		Population	Duration of treatment	Intervention		Outcomes
	T	C	T	C	T	C		(weeks)	Treatment	Control	
Fu et al., 2023	61	61	9.77 ± 2.16	9.52 ± 2.33	29 / 32	31 / 30	Children	4 weeks	(1) XJ capsules, 2–4 capsules per dose, three times daily; (2) Cetirizine Hydrochloride Drops, 1 mL once daily.	Cetirizine Hydrochloride Drops, 1 mL once daily.	①②④⑧
Wang et al., 2022	52	52	36.12 ± 8.59	35.43 ± 9.12	31 / 21	28 / 24	Adult	4 weeks	(1) XJ capsules, 3 capsules per dose, three times daily; (2) Budesonide Nasal Spray, 2 sprays per day.	Budesonide Nasal Spray, 2 sprays per day.	①②⑧
Zhang, 2020	36	36	42.71 ± 6.82	42.48 ± 6.51	20 / 16	21 / 15	Adult	4 weeks	(1) XJ capsules, 4 capsules per dose, three times daily; (2) Montelukast Sodium, 10 mg once daily.	Montelukast Sodium, 10 mg once daily for 28 days.	①②④⑤⑥
Yang et al., 2019	48	48	9.23 ± 1.40	9.14 ± 1.51	30 / 18	32 / 16	Children	4 weeks	(1) XJ capsules, 4 capsules per dose, three times daily; (2) Oxymetazoline Hydrochloride Nasal Spray, once per nostril, twice daily	Oxymetazoline Hydrochloride Nasal Spray, once per nostril, twice daily	①②③④⑤⑥⑦⑧
He et al., 2019	36	36	70.36 ± 9.47	68.99 ± 8.82	18 / 18	20 / 16	Adult	2 weeks	(1) XJ capsules, 4 capsules per dose, three times daily; (2) Fluticasone Propionate Nasal Spray, 200 μg per day	Fluticasone Propionate Nasal Spray, 200 μg per day	①②③⑧
Yang et al., 2018	63	63	36.01 ± 4.04	35.16 ± 4.18	30 / 33	31 / 32	Adult	4 weeks	(1) XJ capsules, 4 capsules per dose, three times daily; (2) Budesonide Nasal Spray, 64 μg per nostril, twice daily.	(1) Loratadine Tablets, 10 mg once daily; (2) Budesonide Nasal Spray, 64 μg per nostril, twice daily	①⑤⑥⑦⑧
Wang et al., 2018	39	39	31.9 ± 5.8	31.4 ± 5.6	22 / 16	21 / 18	Adult	2 weeks	(1) XJ capsules, 4 capsules per dose, three times daily; (2) Budesonide Nasal Spray, 1–2 sprays per nostril, twice daily.	Budesonide Nasal Spray, 1–2 sprays per nostril, twice daily	①⑧
Zhang et al., 2016	35	35	35.2 ± 3.7	35.5 ± 3.2	20 / 15	19 / 16	Adult	4 weeks	(1) XJ capsules, 3 capsules per dose, three times daily; (2) Levocetirizine Hydrochloride Tablets, 5 mg once daily; (3) Budesonide Nasal Spray, 1–2 sprays per nostril, twice daily	(1) Levocetirizine Hydrochloride Tablets, 5 mg once daily; (2) Budesonide Nasal Spray, 1–2 sprays per nostril, twice daily	②④
Zhang et al., 2026	108	108	45.55 ± 1.32 (overall)	45.55 ± 1.32 (overall)	109/107 (overall)	109/107 (overall)	Adult	4 weeks	(1) XJ capsules, 3 capsules per dose, three times daily; (2) Azelastine Hydrochloride Nasal Spray, 1 spray per nostril, twice daily.	Azelastine Hydrochloride Nasal Spray, 1 spray per nostril, twice daily	①②③④⑤⑥⑦⑧
Liang et al., 2016	55	55	9–60	10–62	28 / 27	30 / 25	Children+Adult	6 weeks	(1) XJ capsules, oral administration;(2) Loratadine Tablets, 10 mg once daily for 1 week; (3) Triamcinolone Acetonide Nasal Spray, once daily for 1 week;	(1) Loratadine Tablets, 10 mg once daily for 1 week; (2) Triamcinolone Acetonide Nasal Spray, once daily for 1 week;	①
Sun et al., 2015	35	32	45.28 ± 0.39	45.28 ± 0.39	18 / 17	17 / 15	Adult	4 weeks	(1) XJ capsules, 4 capsules per dose, three times daily; (2) Loratadine, 10 mg once daily; (3) Budesonide Nasal Spray, 1 spray per nostril twice daily	(1) Loratadine, 10 mg once daily; (2) Budesonide Nasal Spray, 1 spray per nostril twice daily	①②③④
Cao et al., 2014	30	30	2–14	2–14	18/12	18/12	Children	10 days	(1) XJ capsules, 3 capsules per dose, twice daily; (2) Loratadine Syrup, 5 mL once daily.	Loratadine Syrup, 5 mL once daily	①
Shi et al., 2012	120	120	15–60	16–63	68 / 52	66 / 54	Adult	6 weeks	(1) XJ capsules, oral administration; (2) Ebastine Tablets, 10 mg once daily; (3) Triamcinolone Acetonide Nasal Spray	(1) Ebastine Tablets, 10 mg once daily; (2) Triamcinolone Acetonide Nasal Spray, once daily	①
Shi et al., 2010	110	110	18–70	18–68	62 / 48	67 / 43	Adult	4 weeks	(1) XJ capsules, 3 capsules per dose (0.3 g per capsule), three times daily; (2) Loratadine, 10 mg once daily	Loratadine, 10 mg once daily	①

Abbreviations: T, treatment group; C, control group; XJ, Xiangju; AR, allergic rhinitis; IL-4, interleukin-4; IL-12, interleukin-12; IgE, immunoglobulin E.

Outcome indicators: ① overall effective rate; ② IL-4; ③ IL-12; ④ IgE; ⑤ runny nose score; ⑥ sneezing score; ⑦ nasal congestion score; ⑧ adverse events.

### Quality evaluation

**FIGURE 2:**
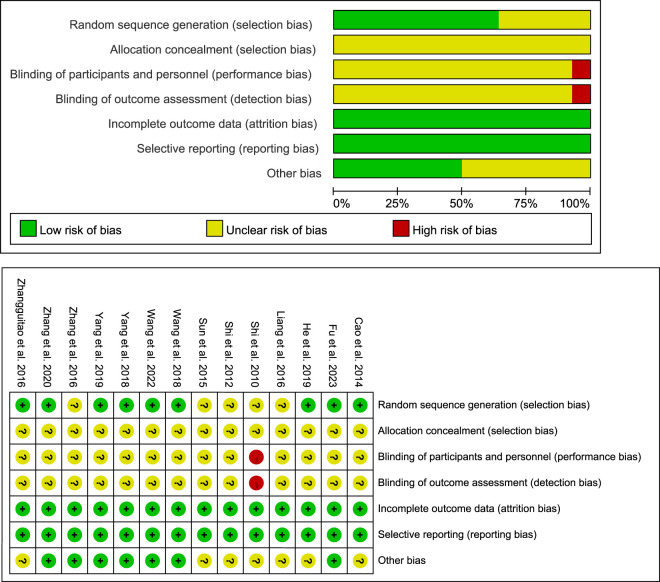
Risk of bias assessment of included randomized controlled trials.

We assessed the quality of 14 RCTs. As shown in Figure 2, important methodological concerns remained in several key domains, particularly allocation concealment and blinding. Adequate random sequence generation was described in 9 studies, indicating a low risk of bias. Five studies mentioned randomization but did not explicitly report the randomization method used, leading to an unclear risk assessment. All studies failed to clearly describe allocation concealment, leading to an unclear risk assessment. Regarding blinding of participants, investigators, and outcome assessors, 13 studies mentioned blinding but provided insufficient details, leading to an unclear risk assessment. One study did not mention blinding and was judged to have a high risk. All studies performed well in terms of incomplete outcome data and selective reporting, with no significant follow-up loss or outcome omission identified. Regarding other bias domains, 7 studies were assessed as unclear risk due to insufficient details on outcome measurement methods or inadequate reporting of patient compliance. The remaining studies were evaluated as low risk. Overall, although all included studies were described as randomized trials, reporting of key methodological details, particularly allocation concealment and blinding, was insufficient. As a result, some risk of bias cannot be excluded, and the efficacy findings should be interpreted cautiously, especially for subjective outcomes.

### Overall effective rate

**FIGURE 3:**
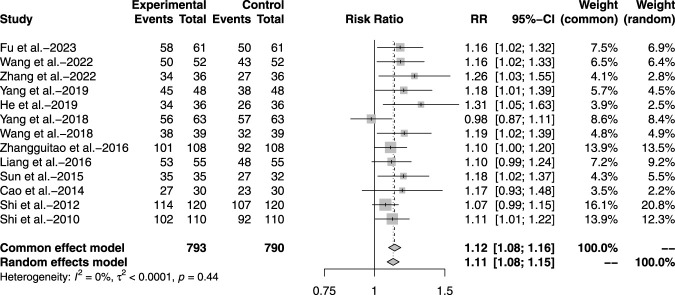
Forest plot of overall effective rate.

As shown in Figure 3, 13 studies used the total effective rate as the primary outcome. A total of 1,583 AR patients were included, and results demonstrated that the clinical effective rate in the XJ capsules group consistently exceeded that of the control group. Meta-analysis using a fixed-effect model showed a higher reported overall effective rate in the XJ capsules group than in the control group (RR = 1.12, 95% CI: 1.08–1.16). Study heterogeneity was low (*I* ^*2*^ = 0%, *p* = 0.44), indicating high consistency of effects across studies. Sensitivity analyses were performed to further validate the robustness of findings. As shown in Supplementary Figure S1, the pooled effect size remained virtually unchanged after sequentially excluding individual studies, confirming the stability of findings.

### IL-4

**FIGURE 4:**
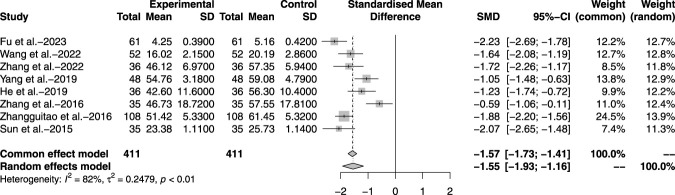
Forest plot of interleukin-4 (IL-4) levels.

As shown in Figure 4, 8 studies used IL-4 levels as the outcome measure, involving 822 participants. Meta-analysis utilizing a random-effects model showed lower IL-4 levels in the XJ capsules group than in the control group (SMD = −1.55, 95% CI: −1.93 to −1.16). However, substantial heterogeneity existed among studies (*I* ^*2*^ = 82%, p < 0.01). As shown in Supplementary Figure S2, sensitivity analysis indicates that the overall results remain robust.

### IL-12

**FIGURE 5:**
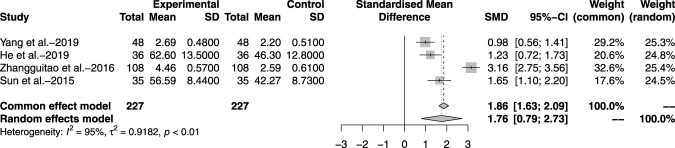
Forest plot of interleukin-12 (IL-12) levels.

A total of 4 studies included IL-12 levels as one of the outcome measures, involving 454 AR participants (Figure 5). Meta-analysis using a random-effects model showed higher IL-12 levels in the XJ capsules group than in the control group (SMD = 1.76, 95% CI: 0.79–2.73). However, substantial heterogeneity existed between studies (*I* ^*2*^ = 95%, *p* < 0.01). As shown in the Supplementary Figure S3, sensitivity analyses revealed no change in effect direction, and all recalculated effect sizes remained statistically significant, confirming the stability of the results.

### IgE

**FIGURE 6:**
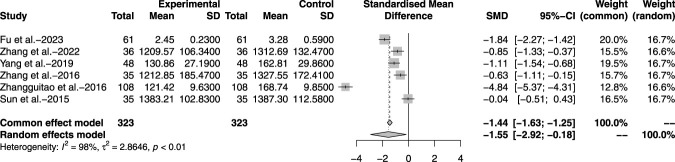
Forest plot of immunoglobulin E (IgE) levels.

As shown in Figure 6, a total of 6 studies assessed serum IgE levels as an outcome measure, involving 646 participants in total. Meta-analysis utilizing a random-effects model showed lower IgE levels in the XJ capsules group compared to the control group (SMD = −1.55, 95% CI: −2.92 to −0.18) with substantial heterogeneity among the included studies (*I* ^*2*^ = 98%, *p* < 0.01). As shown in Supplementary Figure S4, sensitivity analyses revealed changes in effect size but consistent effect direction and statistical significance, suggesting that the direction of the effect on IgE levels was generally stable.

### Runny nose symptom score

**FIGURE 7:**
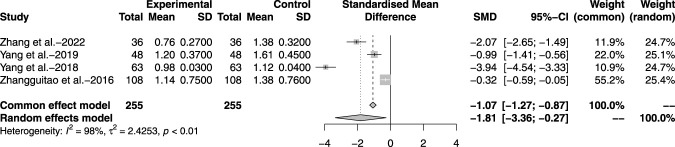
Forest plot of runny nose symptom scores.

Among the included studies, four trials reported runny nose scores before and after treatment with XJ capsules (Figure 7). These studies enrolled a total of 510 AR participants. Analysis revealed significant heterogeneity across studies, indicating substantial inconsistency in outcome measures (*I* ^*2*^ = 98%, *p* < 0.01). Sensitivity analysis using leave-one-out method indicated that heterogeneity remained high even after excluding individual studies (Supplementary Figure S5). Factors such as subjective scoring, inconsistent measurement scales, and varying age groups of included populations may contribute to this high heterogeneity. This study employed a random-effects model for meta-analysis. Pooled results showed lower runny nose symptom scores in the XJ capsules group than in the control group (SMD = −1.81, 95% CI: −3.36 to −0.27).

### Sneezing symptom score

**FIGURE 8:**
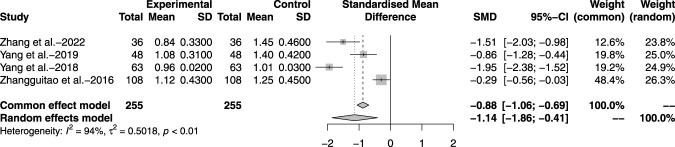
Forest plot of sneezing symptom scores.

Among the included studies, four studies reported sneezing scores before and after treatment with XJ capsules (Figure 8). These studies collectively enrolled 510 patients with AR. The analysis revealed substantial heterogeneity among the included studies (*I* ^*2*^ = 94%, *p* < 0.01). Sensitivity analysis indicated that heterogeneity remained high even after removing individual studies (Supplementary Figure S6). Factors such as subjective scoring, inconsistent measurement scales, and differences in subject characteristics may have contributed to this heterogeneity. Therefore, a random-effects model was used for the meta-analysis. The pooled results showed lower sneezing symptom scores in the XJ capsules group (SMD = −1.14, 95% CI: −1.86 to −0.41).

### Nasal congestion symptom score

**FIGURE 9:**
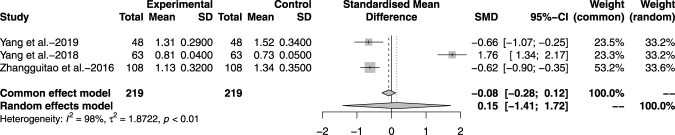
Forest plot of nasal congestion symptom scores.

Among the included studies, three studies reported nasal congestion symptom scores before and after treatment with XJ capsules, involving a total of 438 participants. As shown in Figure 9, the random-effects model revealed no statistically significant difference in nasal congestion scores between the XJ capsules group and the control group (SMD = 0.15, 95% CI: −1.41 to 1.72). The included studies exhibited substantial heterogeneity (*I* ^*2*^ = 98%, *p* < 0.01), indicating substantial inconsistency in effect estimates. Sensitivity analyses after leave-one-out approach showed no statistically significant pooled effect (Supplementary Figure S7). Current evidence is insufficient to support a clear therapeutic effect of XJ capsules in improving nasal congestion symptoms.

### Adverse events

**FIGURE 10:**
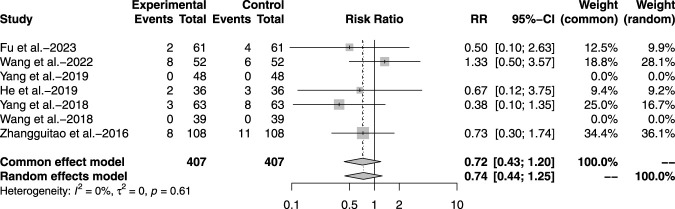
Forest plot of adverse events.

A total of 7 studies reported adverse events during treatment with XJ capsules and control interventions (Figure 10). Meta-analysis performed using a fixed-effect model revealed no statistically significant difference in adverse event incidence between the XJ capsules group and the control group (RR = 0.72, 95% CI: 0.43–1.20). No heterogeneity was detected among studies (*I* ^*2*^ = 0%, *p* = 0.61), indicating consistent safety outcomes across trials. Sensitivity analyses confirmed robust stability of the pooled effect. The pooled effect estimate remained stable after sequential exclusion of individual studies (Supplementary Figure S8). Based on current evidence, no significant difference in reported adverse event incidence was observed.

### Subgroup analyses

To further assess the robustness of the results, this study conducted subgroup analyses based on different populations. After stratification by age, the pooled overall response rates for both the children (RR = 1.17, 95% CI: 1.07–1.29) and adult (RR = 1.12, 95% CI: 1.08–1.17) groups were statistically significant, and no significant differences were observed between the two subgroups (*p* > 0.05) (Supplementary Figure S9). These findings indicate that XJ capsules demonstrate consistent efficacy across different populations. Regarding IL-12 outcomes, both the children group (SMD = 0.98, 95% CI: 0.56–1.41) and the adult group (SMD = 2.02, 95% CI: 0.86–3.18) demonstrated significant increases (Supplementary Figure S10). The test for subgroup differences was not statistically significant (*p* > 0.05), suggesting that age did not significantly influence the effect of XJ capsules on IL-12 levels. Regarding IL-4 outcomes, both the children group (SMD = −1.64, 95% CI: −2.79 to −0.49) and the adult group (SMD = −1.52, 95% CI: −1.94 to −1.09) demonstrated significant reductions (Supplementary Figure S11). The test for subgroup differences was not significant (*p* > 0.05), suggesting that XJ capsules exhibit consistent immunomodulatory effects across different age groups. In subgroup analyses stratified by population, XJ capsules reduced serum IgE levels in the children group (SMD = −1.48, 95% CI: −2.19 to −0.76) (Supplementary Figure S12). In the adult group, the pooled effect also favored XJ capsules, but was not statistically significant (SMD = −1.59, 95% CI: −3.74 to 0.56). Difference tests revealed no significant disparity between the two groups (*p* > 0.05), indicating insufficient evidence to conclude that age modulates XJ capsules’ efficacy in reducing IgE levels. Overall, XJ capsules demonstrates a certain advantage in lowering IgE levels, with more robust evidence in pediatric populations. Findings in adults require further validation through high-quality studies. Regarding symptom outcomes, subgroup analyses stratified by population showed that XJ capsules reduced runny nose scores in both the children group (SMD = −0.99, 95% CI: −1.41 to −0.56) and the adult group (SMD = −2.09, 95% CI: −4.15 to −0.04) (Supplementary Figure S13), reduced sneezing scores in both the children group (SMD = −0.86, 95% CI: −1.28 to −0.44) and the adult group (SMD = −1.24, 95% CI: −2.22 to −0.25) (Supplementary Figure S14), and showed no significant subgroup difference for either outcome (p > 0.05). For nasal congestion, the children subgroup showed a significant reduction (SMD = −0.66, 95% CI: −1.07 to −0.25), whereas the adult subgroup did not show a significant difference (SMD = 0.56, 95% CI: −1.77 to 2.89) (Supplementary Figure S15). However, the test for subgroup differences was not statistically significant (*p* > 0.05), suggesting that age was not a significant source of heterogeneity for these symptom outcomes. In subgroup analyses stratified by population, no significant difference in adverse event incidence was observed in either the children group (RR = 0.50, 95% CI: 0.10–2.63) or the adult group (RR = 0.77, 95% CI: 0.44–1.34) (Supplementary Figure S16). The test for subgroup differences was not statistically significant (*p* > 0.05), suggesting that age did not significantly influence the safety outcome of XJ capsules.

Subgroup analyses were conducted based on different combination regimens. Regarding the overall effective rate, the effect size for the antihistamine combination regimen was RR = 1.12 (95% CI: 1.06–1.19), the RR for the nasal corticosteroid combination regimen was 1.13 (95% CI: 1.05–1.21), and the RR for other combination regimens was 1.13 (95% CI: 1.07–1.19) (Supplementary Figure S17). No statistically significant subgroup difference was observed among combination regimens. In the analysis of IL-4 outcomes, significant reductions were observed across all treatment subgroups. The greatest reduction was seen with the antihistamine combination regimen (SMD = −1.99, 95% CI: −2.26 to −1.73), followed by the nasal corticosteroid combination regimen (SMD = −1.46, 95% CI: −1.79 to −1.12) and other regimens (SMD = −1.34, 95% CI: −1.98 to −0.69) (Supplementary Figure S18). Notably, heterogeneity was markedly reduced in the antihistamine (*I* ^*2*^ = 36%) and nasal corticosteroid (*I* ^*2*^ = 28%) groups, indicating that differences in combination therapy significantly contributed to the heterogeneity of IL-4 outcomes. Under the fixed-effects model, subgroup differences were statistically significant (*p* < 0.01), and under the random-effects model, they approached statistical significance (*p* = 0.05), suggesting that the combination therapy regimen may partially explain the heterogeneity in IL-4 outcomes. In the IgE outcome analysis, XJ capsules demonstrated a significant reduction in IgE levels both in the antihistamine combination regimen (SMD = −3.34, 95% CI: −6.28 to −0.40) and in other combination regimens (SMD = −0.66, 95% CI: −1.11 to −0.21) (Supplementary Figure S19). The difference between the two groups was not statistically significant (*p* > 0.05), suggesting that XJ capsules combined with different treatment regimens can consistently reduce IgE levels in AR patients.

Subgroup analyses were performed according to treatment duration. Regarding the overall effective rate, the ≤2 weeks subgroup (RR = 1.22, 95% CI: 1.09–1.36), the 2–6 weeks subgroup (RR = 1.12, 95% CI: 1.07–1.17), and ≥6 weeks (RR = 1.08, 95% CI: 1.01–1.15) all demonstrated significant clinical improvement with XJ capsules (Supplementary Figure S20). Differences between subgroups were not statistically significant. These results suggest that the clinical efficacy of XJ capsules is consistent across different treatment durations. In the IL-4 analysis, both the 2–6 weeks subgroup (SMD = −1.59, 95% CI: −2.02 to −1.16) and the ≤2 weeks subgroup (SMD = −1.23, 95% CI: −1.74 to −0.72) demonstrated significant reductions (Supplementary Figure S21). Subgroup difference tests failed to reach statistical significance, suggesting that variations in treatment duration do not significantly influence the degree of IL-4 reduction.

Overall, subgroup analyses showed a generally consistent direction of effect across different populations, combination regimens, and treatment durations. Combination therapy partially explains the heterogeneity observed in IL-4 outcomes. These findings support the stability of the observed direction of effect, but do not fully explain the heterogeneity across outcomes.

### Publication bias assessment

**FIGURE 11:**
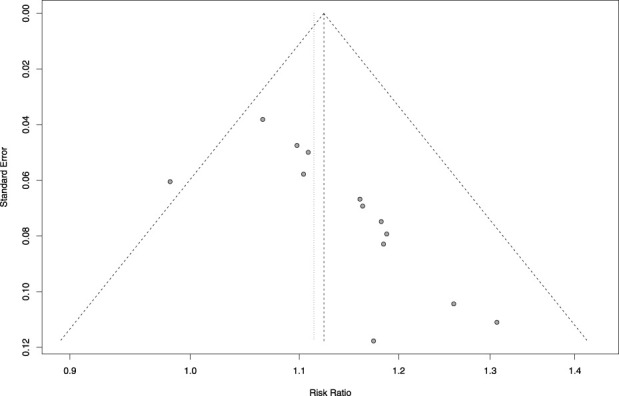
Funnel plot for publication bias assessment of overall effective rate.

A publication bias analysis was performed for outcomes that included ten or more studies. For the overall effective rate outcome, funnel plots exhibited marked asymmetry (Figure 11), suggesting potential publication bias. This was further supported by Egger’s test, which identified significant small-study effects (*t* = 3.49, *p* = 0.005). The Begg’s rank correlation test yielded consistent results (*z* = 3.05, *p* = 0.002), providing additional evidence of publication bias. After applying trim-and-fill methods, five potentially missing studies were imputed (Supplementary Figure S22). The adjusted pooled effect size decreased slightly (RR = 1.09, 95% CI: 1.06–1.13) but remained statistically significant. This indicates that although the original effect may have been slightly overestimated, the directionality and robustness of the results were not substantially affected.

### Certainty assessment

According to the GRADE evidence assessment system (Supplementary Table S4), the quality of evidence for each outcome ranged from moderate to very low. The primary efficacy outcome was rated as low certainty, whereas most other efficacy outcomes were rated as very low certainty. The primary reasons for downgrading included risk of bias in studies, substantial heterogeneity between studies, and publication bias for some outcomes. Although most outcomes supported the efficacy of XJ capsules, caution is warranted in interpretation.

## Discussion

This meta-analysis synthesized evidence from 14 RCTs involving 1,968 patients with AR to evaluate the efficacy and safety of XJ capsules as an adjunct to conventional therapy. Compared with conventional therapy alone, XJ capsules combined with conventional therapy were associated with a higher reported overall effective rate and favorable changes in several symptom and immunological outcomes. In addition, XJ capsules were associated with lower serum IL-4 and IgE levels and higher IL-12 levels, suggesting a potential immunomodulatory effect. Symptom-based results showed lower runny nose and sneezing scores, whereas no clear benefit was observed for nasal congestion. The adverse event results indicate no significant difference in the incidence of adverse reactions among the groups, suggesting that the addition of XJ capsules does not increase treatment-related adverse reactions. In conclusion, these findings suggest that XJ capsules may provide additional clinical and immunologic benefits for AR when used alongside conventional pharmacotherapy, but the results should be interpreted cautiously because of methodological limitations and low certainty of evidence.

The findings of this meta-analysis suggest that XJ capsules combined with conventional treatment may improve the reported overall effective rate, alleviate some nasal symptoms, and modulate immune-related markers. Consistent with our results, a Meta-analysis based on 23 studies by Chen et al. (2023) indicated that the combination of traditional Chinese medicine with conventional treatment was superior to biomedicine alone in relieving AR symptoms and regulating immune function, and that the combination treatment could more effectively inhibit Th2-associated immune factors (IL-4, IgE) with a higher safety profile. A meta-analysis by Zhang et al. (2023) that included seven studies found that oral TCM treatment in AR patients significantly reduced the recurrence rate, and that the quality of life of patients taking oral TCM after treatment was significantly higher than that of the control group. Nevertheless, although there is a growing body of evidence supporting the clinical efficacy of traditional Chinese medicine therapies as adjunctive treatments for AR, there is no meta-analysis evaluating the efficacy and safety of XJ capsules for the treatment of AR. This study represents the first systematic evaluation of the efficacy and safety of XJ capsules for AR, offering targeted clinical evidence to inform their use as an adjunctive therapy in AR.

The therapeutic effects of XJ capsules may be related to the combined actions of multiple botanical drugs and their reported metabolites. XJ capsules are a standardized multi-botanical proprietary Chinese medicine formula. Existing pharmacological studies suggest that several botanical drugs in this formulation may contribute to anti-allergic and immunomodulatory effects. Astragali Radix may exert therapeutic effects on AR by modulating inflammatory and immune pathways through multi-targets, especially by inhibiting the NF-κB and TNF signaling cascade, reducing the production of pro-inflammatory cytokines, and attenuating IL-13-induced inflammation in the nasal mucosa (Hua et al., 2024; He et al., 2022; Guo and Xu, 2021). Saposhnikoviae Radix-derived extracts can reduce the local inflammatory response of nasal mucosa by modulating the TLR4/NF-κB signaling pathway (Chen et al., 2022). They may also reduce IgE and histamine levels and inhibit IgE-induced mast cell degranulation, thus inhibiting allergic reactions (Li et al., 2022). Magnoliae Flos contains volatile metabolites that inhibit histamine release and mast cell activation, reduce pro-inflammatory cytokines, and attenuate type I hypersensitivity and related inflammatory cascade reactions (Dong et al., 2025). In addition, Prunellae Spica may inhibit mast cell-mediated allergic responses and reduce serum histamine release (Kim et al., 2007). Glycyrrhizae Radix et Rhizoma contains glycyrrhizic acid, which can regulate allergy-related immune cells and inhibit IgE-mediated allergic reactions (Han et al., 2017). Angelicae Dahuricae Radix is rich in coumarin metabolites, which have anti-allergic and anti-inflammatory effects by reducing histamine release and inflammatory cytokine production (Li and Wu, 2017). Chrysanthemi Indici Flos has anti-inflammatory and immunomodulatory activity (Cheng et al., 2005). Chuanxiong Rhizoma contains tetramethylpyrazine and other metabolites, which may regulate Th1/Th2 and Treg/Th17 imbalance in experimental allergic airway inflammation (Ji et al., 2014). These findings suggest that the effects of XJ capsules may not depend on a single botanical drug alone, but rather on the complementary actions of multiple botanical drugs. Consistent with this interpretation, network pharmacology evidence on Xiang Ju preparations identified CXCL8, IL1B, IL6, IL10, TNF, IL4, IL13, IL5, ICAM1, and MMP9 as relevant AR-related targets (Hu et al., 2019). These targets are involved in inflammatory-cell recruitment, cytokine amplification, Th2-skewed immune responses, IgE production, eosinophil migration, immune regulation, and mucosal remodeling. Therefore, XJ capsules may exert formula-level effects through coordinated regulation of multiple inflammatory and immune pathways. This pharmacological profile provides a plausible explanation for the reduction in IL-4 and IgE levels, the increase in IL-12 levels, and the improvement in some clinical symptoms observed in this meta-analysis.

This study showed that XJ capsules combined with conventional treatment reduced the symptom scores of sneezing and runny nose, which may be closely related to the inhibition of histamine release and reduction of inflammatory mediator levels by XJ capsules. In contrast, nasal congestion symptoms did not show significant improvement. These findings may be explained by the small number of included studies and the high degree of heterogeneity. It may also reflect the fact that the symptom of nasal congestion is more related to vasodilatation and mucosal structural changes, which are limited to be improved by XJ capsules. Clinically, nasal congestion is usually more responsive to nasal glucocorticoids or decongestants (Storms, 2008; Xu et al., 2020), which may partly explain why this outcome did not reach statistical significance. In this study, XJ capsules were found to reduce the levels of serum IgE and IL-4, and elevate the level of IL-12. IL-4, a typical Th2-type cytokine, promotes B-cell IgE class switching, which is a key driver in the development of AR (Nur Husna et al., 2022; Quan and Zhang, 2025). And IL-12, as a Th1-type cytokine, can antagonize the Th2 response and promote immune tolerance (Gavett et al., 1995; Wills-Karp, 2001). The bidirectional regulation of these indicators by XJ capsules suggests that it may improve the pathology of AR by correcting the Th1/Th2 immune imbalance. Safety results indicate that there was no significant difference in the incidence of reported adverse events between the XJ capsule group and the control group, suggesting that the drug is generally well tolerated in the short term. According to official product information, reported adverse reactions primarily involved gastrointestinal, skin, and neurological symptoms. However, in the included studies, broader safety aspects such as long-term adverse reactions and drug interactions were not systematically evaluated.

To the best of our knowledge, this is the first meta-analysis to systematically evaluate the efficacy and safety of XJ capsules for AR. This study integrated clinical outcomes and immunological indexes, and performed multi-group subgroup analysis, which provided more comprehensive evidence than previous studies, and provided an important reference for its clinical application. Nonetheless, several limitations should be noted. First, the methodological quality of the included trials was limited. None of the studies explicitly reported on allocation concealment, and some trials did not provide a sufficient description of the blinding procedures. These limitations may introduce important bias, particularly for subjective outcomes, and should be considered when interpreting the results. Second, there was high heterogeneity in some continuous variable outcomes (IL-4, IL-12, IgE, and symptom scores), which may be related to differences in testing methods, patient populations, and coadministration. Although the direction of effect was generally consistent, this very serious inconsistency reduces confidence in the reliability of the pooled effect sizes, particularly the magnitude of the estimated benefit. Third, all included studies were conducted in China, which may limit the external validity of the findings for other ethnic groups and healthcare systems. Fourth, the primary outcome remained statistically significant after correction by the trim-and-fill method, but the effect size was reduced, suggesting that the original results may have been somewhat overestimated. Fifth, the included studies did not consistently use standardized symptom assessment tools, such as TNSS, which may reduce comparability across studies and affect the interpretability of pooled symptom outcomes. In addition, there are currently no validated and universally applicable Minimal Clinically Important Difference (MCID) thresholds for the symptom outcomes reported in the included studies. Although some pooled effects are statistically significant, their clinical significance cannot yet be formally established. Finally, safety evidence remains limited, as most trials provided only brief adverse event reporting and did not systematically assess long-term adverse reactions, drug interactions, or other safety outcomes. In conclusion, although the quality of the existing evidence is limited, this study suggests the potential value of XJ capsules as an adjunct to conventional treatment in AR. Further evidence from well-designed, multicenter, large-scale RCTs using standardized outcome measures is needed to confirm these findings and clarify the role of XJ capsules in AR management.

## Conclusion

This meta-analysis suggests that XJ capsules may provide additional benefits for some symptom and immunologic outcomes in patients with AR. No significant increase in reported adverse events was observed. These findings suggest that XJ capsules may be an effective adjunctive therapy for the treatment of AR. However, methodological limitations in the current evidence suggest that these results should be interpreted with caution. Well-designed, large, multicenter, RCTs employing standardized outcome measures are needed to further confirm these results and to clarify the role of XJ capsules in routine clinical practice.

## Data Availability

The raw data supporting the conclusions of this article will be made available by the authors, without undue reservation.
