# Supplementary material for: Efficacy and safety of Xiangju capsules for allergic rhinitis: a systematic review and meta-analysis of randomized controlled trials
Source: Front Pharmacol. 2026 May 28;17:1788708. doi: 10.3389/fphar.2026.1788708 (PMC13254496; doi:10.3389/fphar.2026.1788708)

**Supplementary Material**

Supplementary Table 1. Detailed PRISMA checklist.

| **Section and Topic** | **Item #** | **Checklist item** | **Location** |
| --- | --- | --- | --- |
| **TITLE** | | |  |
| Title | 1 | Identify the report as a systematic review. | Title |
| **ABSTRACT** | | |  |
| Abstract | 2 | See the PRISMA 2020 for Abstracts checklist. | Abstract |
| **INTRODUCTION** | | |  |
| Rationale | 3 | Describe the rationale for the review in the context of existing knowledge. | Introduction |
| Objectives | 4 | Provide an explicit statement of the objective(s) or question(s) the review addresses. | Introduction |
| **METHODS** | | |  |
| Eligibility criteria | 5 | Specify the inclusion and exclusion criteria for the review and how studies were grouped for the syntheses. | Methods: Inclusion criteria; Exclusion criteria |
| Information sources | 6 | Specify all databases, registers, websites, organisations, reference lists and other sources searched or consulted to identify studies. Specify the date when each source was last searched or consulted. | Methods: Data sources and search strategy |
| Search strategy | 7 | Present the full search strategies for all databases, registers and websites, including any filters and limits used. | Supplementary Table 2 |
| Selection process | 8 | Specify the methods used to decide whether a study met the inclusion criteria of the review, including how many reviewers screened each record and each report retrieved, whether they worked independently, and if applicable, details of automation tools used in the process. | Methods: Study selection and data extraction |
| Data collection process | 9 | Specify the methods used to collect data from reports, including how many reviewers collected data from each report, whether they worked independently, any processes for obtaining or confirming data from study investigators, and if applicable, details of automation tools used in the process. | Methods: Study selection and data extraction |
| Data items | 10a | List and define all outcomes for which data were sought. Specify whether all results that were compatible with each outcome domain in each study were sought (e.g. for all measures, time points, analyses), and if not, the methods used to decide which results to collect. | Methods: Inclusion criteria; Study selection and data extraction |
|  | 10b | List and define all other variables for which data were sought (e.g. participant and intervention characteristics, funding sources). Describe any assumptions made about any missing or unclear information. | Methods: Study selection and data extraction; Results: Characteristics of the included studies |
| Study risk of bias assessment | 11 | Specify the methods used to assess risk of bias in the included studies, including details of the tool(s) used, how many reviewers assessed each study and whether they worked independently, and if applicable, details of automation tools used in the process. | Methods: Risk of bias assessment |
| Effect measures | 12 | Specify for each outcome the effect measure(s) (e.g. risk ratio, mean difference) used in the synthesis or presentation of results. | Methods: Statistical analysis |
| Synthesis methods | 13a | Describe the processes used to decide which studies were eligible for each synthesis (e.g. tabulating the study intervention characteristics and comparing against the planned groups for each synthesis (item #5)). | Methods: Statistical analysis |
|  | 13b | Describe any methods required to prepare the data for presentation or synthesis, such as handling of missing summary statistics, or data conversions. | Methods: Statistical analysis |
|  | 13c | Describe any methods used to tabulate or visually display results of individual studies and syntheses. | Methods: Statistical analysis; Results |
|  | 13d | Describe any methods used to synthesize results and provide a rationale for the choice(s). If meta-analysis was performed, describe the model(s), method(s) to identify the presence and extent of statistical heterogeneity, and software package(s) used. | Methods: Statistical analysis |
|  | 13e | Describe any methods used to explore possible causes of heterogeneity among study results (e.g. subgroup analysis, meta-regression). | Methods: Statistical analysis; Results: Subgroup analyses |
|  | 13f | Describe any sensitivity analyses conducted to assess robustness of the synthesized results. | Methods: Statistical analysis; Results |
| Reporting bias assessment | 14 | Describe any methods used to assess risk of bias due to missing results in a synthesis (arising from reporting biases). | Methods: Statistical analysis; Results: Publication bias assessment |
| Certainty assessment | 15 | Describe any methods used to assess certainty (or confidence) in the body of evidence for an outcome. | Methods: Quality of evidence |
| **RESULTS** | | |  |
| Study selection | 16a | Describe the results of the search and selection process, from the number of records identified in the search to the number of studies included in the review, ideally using a flow diagram. | Results: Study selection; Figure 1 |
|  | 16b | Cite studies that might appear to meet the inclusion criteria, but which were excluded, and explain why they were excluded. | Results: Study selection |
| Study characteristics | 17 | Cite each included study and present its characteristics. | Results: Characteristics of the included studies; Table 1 |
| Risk of bias in studies | 18 | Present assessments of risk of bias for each included study. | Results: Quality evaluation; Figure 2 |
| Results of individual studies | 19 | For all outcomes, present, for each study: (a) summary statistics for each group (where appropriate) and (b) an effect estimate and its precision (e.g. confidence/credible interval), ideally using structured tables or plots. | Results; Figures 3–10 |
| Results of syntheses | 20a | For each synthesis, briefly summarise the characteristics and risk of bias among contributing studies. | Results |
|  | 20b | Present results of all statistical syntheses conducted. If meta-analysis was done, present for each the summary estimate and its precision (e.g. confidence/credible interval) and measures of statistical heterogeneity. If comparing groups, describe the direction of the effect. | Results; Figures 3–10 |
|  | 20c | Present results of all investigations of possible causes of heterogeneity among study results. | Results: Subgroup analyses |
|  | 20d | Present results of all sensitivity analyses conducted to assess the robustness of the synthesized results. | Results; Supplementary Figures 1–8 |
| Reporting biases | 21 | Present assessments of risk of bias due to missing results (arising from reporting biases) for each synthesis assessed. | Results: Publication bias assessment; Figure 11 |
| Certainty of evidence | 22 | Present assessments of certainty (or confidence) in the body of evidence for each outcome assessed. | Results: Certainty assessment; Supplementary Table 4 |
| **DISCUSSION** | | |  |
| Discussion | 23a | Provide a general interpretation of the results in the context of other evidence. | Discussion |
|  | 23b | Discuss any limitations of the evidence included in the review. | Discussion |
|  | 23c | Discuss any limitations of the review processes used. | Discussion |
|  | 23d | Discuss implications of the results for practice, policy, and future research. | Discussion |
| **OTHER INFORMATION** | | |  |
| Registration and protocol | 24a | Provide registration information for the review, including register name and registration number, or state that the review was not registered. | Methods |
|  | 24b | Indicate where the review protocol can be accessed, or state that a protocol was not prepared. | Methods |
|  | 24c | Describe and explain any amendments to information provided at registration or in the protocol. | Not applicable |
| Support | 25 | Describe sources of financial or non-financial support for the review, and the role of the funders or sponsors in the review. | Funding Statement |
| Competing interests | 26 | Declare any competing interests of review authors. | Conflicts of Interest |
| Availability of data, code and other materials | 27 | Report which of the following are publicly available and where they can be found: template data collection forms; data extracted from included studies; data used for all analyses; analytic code; any other materials used in the review. | Data Availability |

*From:*  Page MJ, McKenzie JE, Bossuyt PM, Boutron I, Hoffmann TC, Mulrow CD, et al. The PRISMA 2020 statement: an updated guideline for reporting systematic reviews. BMJ 2021;372:n71. doi: 10.1136/bmj.n71. This work is licensed under CC BY 4.0. To view a copy of this license, visit <https://creativecommons.org/licenses/by/4.0/>

Supplementary Table 2. Search Strategies for Each Database

| **Database** | **Search Criteria** |
| --- | --- |
| **Pubmed** | #1 "Rhinitis, Allergic"[Mesh]  OR "allergic rhinitis"[Title/Abstract]  OR "hay fever"[Title/Abstract]  #2 "Xiangju capsule"[Title/Abstract]  OR "Xiangju capsules"[Title/Abstract]  OR "XJ capsule"[Title/Abstract]  OR "XJ capsules"[Title/Abstract]  #3 randomized controlled trial[Publication Type]  OR random*[Title/Abstract]  OR RCT[Title/Abstract]  #4 #1 AND #2 AND #3 |
| **Embase** | #1 'allergic rhinitis'/exp  OR 'allergic rhinitis':ti,ab  OR 'hay fever':ti,ab  #2 'xiangju capsule':ti,ab  OR 'xiangju capsules':ti,ab  OR 'xj capsule':ti,ab  OR 'xj capsules':ti,ab  #3 'randomized controlled trial'/exp  OR random*:ti,ab  OR rct:ti,ab  #4 #1 AND #2 AND #3 |
| **Cochrane Library** | #1 (allergic rhinitis OR hay fever):ti,ab,kw  #2 (xiangju capsule OR xiangju capsules OR xj capsule OR xj capsules):ti,ab,kw  #3 #1 AND #2 |
| **Web of Science** | TS = (  ("allergic rhinitis" OR "hay fever")  AND  ("xiangju capsule" OR "xiangju capsules" OR "xj capsule" OR "xj capsules")  AND  (random* OR trial* OR RCT)  ) |
| **CNKI** | 主题 = (过敏性鼻炎 OR 变应性鼻炎)  AND  主题 = (香菊胶囊 OR 香菊)  AND  主题 = (  随机  OR 随机对照  OR 随机对照试验  OR 临床试验  OR 临床研究  ) |
| **Wanfang Database** | 主题: (过敏性鼻炎 OR 变应性鼻炎)  AND  主题: (香菊胶囊 OR 香菊)  AND  主题: (  随机  OR 随机对照  OR 随机对照试验  OR 临床试验  OR 临床研究  ) |
| **VIP Database** | 关键词 = (过敏性鼻炎 OR 变应性鼻炎)  AND  关键词 = (香菊胶囊 OR 香菊)  AND  关键词 = (  随机  OR 随机对照  OR 随机对照试验  OR 临床试验  OR 临床研究  ) |
| **CBM** | #1 主题词: 过敏性鼻炎  OR 自由词: 过敏性鼻炎 OR 变应性鼻炎  #2 自由词: 香菊胶囊 OR 香菊  #3 主题词: 随机对照试验  OR 自由词:  随机  OR 随机对照  OR 随机对照试验  OR 临床试验  OR 临床研究  #4 #1 AND #2 AND #3 |

Supplementary Table 3. Composition of Xiangju capsules and availability of formulation details in the included studies

| **Study** | **Year** | **Formulation** | **Composition** | **Source information** |
| --- | --- | --- | --- | --- |
| Fu et al. | 2023 | Xiangju capsules | Huaxiangshu fruit infructescence with seeds removed, Platycarya strobilacea Siebold & Zucc. [Juglandaceae]; Prunella vulgaris L. [Lamiaceae; Prunellae Spica]; Chrysanthemum indicum L. [Asteraceae; Chrysanthemi Indici Flos]; Astragalus mongholicus Bunge [Fabaceae; Astragali Radix]; Magnolia biondii Pamp. [Magnoliaceae; Magnoliae Flos]; Saposhnikovia divaricata (Turcz. ex Ledeb.) Schischk. [Apiaceae; Saposhnikoviae Radix]; Angelica dahurica (Hoffm.) Benth. & Hook.f. ex Franch. & Sav. [Apiaceae; Angelicae Dahuricae Radix]; Glycyrrhiza uralensis Fisch. [Fabaceae; Glycyrrhizae Radix et Rhizoma]; and Ligusticum chuanxiong Hort. [Apiaceae; Chuanxiong Rhizoma]. Maize starch and magnesium stearate are listed as excipients. | The study identified Xiangju capsules as the intervention and reported the manufacturer, specification, and batch number. The full composition was not provided in the original article and was supplemented from product information. |
| Wang et al. | 2022 | Xiangju capsules | Huaxiangshu fruit infructescence with seeds removed, Platycarya strobilacea Siebold & Zucc. [Juglandaceae]; Prunella vulgaris L. [Lamiaceae; Prunellae Spica]; Chrysanthemum indicum L. [Asteraceae; Chrysanthemi Indici Flos]; Astragalus mongholicus Bunge [Fabaceae; Astragali Radix]; Magnolia biondii Pamp. [Magnoliaceae; Magnoliae Flos]; Saposhnikovia divaricata (Turcz. ex Ledeb.) Schischk. [Apiaceae; Saposhnikoviae Radix]; Angelica dahurica (Hoffm.) Benth. & Hook.f. ex Franch. & Sav. [Apiaceae; Angelicae Dahuricae Radix]; Glycyrrhiza uralensis Fisch. [Fabaceae; Glycyrrhizae Radix et Rhizoma]; and Ligusticum chuanxiong Hort. [Apiaceae; Chuanxiong Rhizoma]. Maize starch and magnesium stearate are listed as excipients. | The study identified Xiangju capsules as the intervention and reported the manufacturer, specification, and approval number. The full composition was not reported in the original article and was supplemented from product information. |
| Zhang et al. | 2020 | Xiangju capsules | Huaxiangshu fruit infructescence with seeds removed, Platycarya strobilacea Siebold & Zucc. [Juglandaceae]; Prunella vulgaris L. [Lamiaceae; Prunellae Spica]; Chrysanthemum indicum L. [Asteraceae; Chrysanthemi Indici Flos]; Astragalus mongholicus Bunge [Fabaceae; Astragali Radix]; Magnolia biondii Pamp. [Magnoliaceae; Magnoliae Flos]; Saposhnikovia divaricata (Turcz. ex Ledeb.) Schischk. [Apiaceae; Saposhnikoviae Radix]; Angelica dahurica (Hoffm.) Benth. & Hook.f. ex Franch. & Sav. [Apiaceae; Angelicae Dahuricae Radix]; Glycyrrhiza uralensis Fisch. [Fabaceae; Glycyrrhizae Radix et Rhizoma]; and Ligusticum chuanxiong Hort. [Apiaceae; Chuanxiong Rhizoma]. Maize starch and magnesium stearate are listed as excipients. | The study identified Xiangju capsules as the intervention and reported the approval number. The full composition was not reported in the original article and was supplemented from product information. |
| Yang et al. | 2019 | Xiangju capsules | Huaxiangshu fruit infructescence with seeds removed, Platycarya strobilacea Siebold & Zucc. [Juglandaceae]; Prunella vulgaris L. [Lamiaceae; Prunellae Spica]; Chrysanthemum indicum L. [Asteraceae; Chrysanthemi Indici Flos]; Astragalus mongholicus Bunge [Fabaceae; Astragali Radix]; Magnolia biondii Pamp. [Magnoliaceae; Magnoliae Flos]; Saposhnikovia divaricata (Turcz. ex Ledeb.) Schischk. [Apiaceae; Saposhnikoviae Radix]; Angelica dahurica (Hoffm.) Benth. & Hook.f. ex Franch. & Sav. [Apiaceae; Angelicae Dahuricae Radix]; Glycyrrhiza uralensis Fisch. [Fabaceae; Glycyrrhizae Radix et Rhizoma]; and Ligusticum chuanxiong Hort. [Apiaceae; Chuanxiong Rhizoma]. Maize starch and magnesium stearate are listed as excipients. | The study identified Xiangju capsules as the intervention. Detailed formulation information was not reported in the original article and the full composition was supplemented from product information. |
| He et al. | 2019 | Xiangju capsules | Huaxiangshu fruit infructescence with seeds removed, Platycarya strobilacea Siebold & Zucc. [Juglandaceae]; Prunella vulgaris L. [Lamiaceae; Prunellae Spica]; Chrysanthemum indicum L. [Asteraceae; Chrysanthemi Indici Flos]; Astragalus mongholicus Bunge [Fabaceae; Astragali Radix]; Magnolia biondii Pamp. [Magnoliaceae; Magnoliae Flos]; Saposhnikovia divaricata (Turcz. ex Ledeb.) Schischk. [Apiaceae; Saposhnikoviae Radix]; Angelica dahurica (Hoffm.) Benth. & Hook.f. ex Franch. & Sav. [Apiaceae; Angelicae Dahuricae Radix]; Glycyrrhiza uralensis Fisch. [Fabaceae; Glycyrrhizae Radix et Rhizoma]; and Ligusticum chuanxiong Hort. [Apiaceae; Chuanxiong Rhizoma]. Maize starch and magnesium stearate are listed as excipients. | The study identified Xiangju capsules as the intervention and reported the manufacturer and batch number. The discussion section also mentioned the main ingredients, but the full formulation was not presented in a standardized way. The complete composition was supplemented from product information. |
| Yang et al. | 2018 | Xiangju capsules | Huaxiangshu fruit infructescence with seeds removed, Platycarya strobilacea Siebold & Zucc. [Juglandaceae]; Prunella vulgaris L. [Lamiaceae; Prunellae Spica]; Chrysanthemum indicum L. [Asteraceae; Chrysanthemi Indici Flos]; Astragalus mongholicus Bunge [Fabaceae; Astragali Radix]; Magnolia biondii Pamp. [Magnoliaceae; Magnoliae Flos]; Saposhnikovia divaricata (Turcz. ex Ledeb.) Schischk. [Apiaceae; Saposhnikoviae Radix]; Angelica dahurica (Hoffm.) Benth. & Hook.f. ex Franch. & Sav. [Apiaceae; Angelicae Dahuricae Radix]; Glycyrrhiza uralensis Fisch. [Fabaceae; Glycyrrhizae Radix et Rhizoma]; and Ligusticum chuanxiong Hort. [Apiaceae; Chuanxiong Rhizoma]. Maize starch and magnesium stearate are listed as excipients. | The study identified Xiangju capsules as the intervention, but did not provide detailed formulation information. The full composition was supplemented from product information. |
| Wang et al. | 2018 | Xiangju capsules | Huaxiangshu fruit infructescence with seeds removed, Platycarya strobilacea Siebold & Zucc. [Juglandaceae]; Prunella vulgaris L. [Lamiaceae; Prunellae Spica]; Chrysanthemum indicum L. [Asteraceae; Chrysanthemi Indici Flos]; Astragalus mongholicus Bunge [Fabaceae; Astragali Radix]; Magnolia biondii Pamp. [Magnoliaceae; Magnoliae Flos]; Saposhnikovia divaricata (Turcz. ex Ledeb.) Schischk. [Apiaceae; Saposhnikoviae Radix]; Angelica dahurica (Hoffm.) Benth. & Hook.f. ex Franch. & Sav. [Apiaceae; Angelicae Dahuricae Radix]; Glycyrrhiza uralensis Fisch. [Fabaceae; Glycyrrhizae Radix et Rhizoma]; and Ligusticum chuanxiong Hort. [Apiaceae; Chuanxiong Rhizoma]. Maize starch and magnesium stearate are listed as excipients. | The study identified Xiangju capsules as the intervention and reported the manufacturer, specification, and batch number. The full composition was not reported in the original article and was supplemented from product information. |
| Zhang et al. | 2016 | Xiangju capsules | Huaxiangshu fruit infructescence with seeds removed, Platycarya strobilacea Siebold & Zucc. [Juglandaceae]; Prunella vulgaris L. [Lamiaceae; Prunellae Spica]; Chrysanthemum indicum L. [Asteraceae; Chrysanthemi Indici Flos]; Astragalus mongholicus Bunge [Fabaceae; Astragali Radix]; Magnolia biondii Pamp. [Magnoliaceae; Magnoliae Flos]; Saposhnikovia divaricata (Turcz. ex Ledeb.) Schischk. [Apiaceae; Saposhnikoviae Radix]; Angelica dahurica (Hoffm.) Benth. & Hook.f. ex Franch. & Sav. [Apiaceae; Angelicae Dahuricae Radix]; Glycyrrhiza uralensis Fisch. [Fabaceae; Glycyrrhizae Radix et Rhizoma]; and Ligusticum chuanxiong Hort. [Apiaceae; Chuanxiong Rhizoma]. Maize starch and magnesium stearate are listed as excipients. | The study identified Xiangju capsules as the intervention and reported the manufacturer and approval number. The full composition was not reported in the original article and was supplemented from product information. |
| Zhangguitao et al. | 2016 | Xiangju capsules | Huaxiangshu fruit infructescence with seeds removed, Platycarya strobilacea Siebold & Zucc. [Juglandaceae]; Prunella vulgaris L. [Lamiaceae; Prunellae Spica]; Chrysanthemum indicum L. [Asteraceae; Chrysanthemi Indici Flos]; Astragalus mongholicus Bunge [Fabaceae; Astragali Radix]; Magnolia biondii Pamp. [Magnoliaceae; Magnoliae Flos]; Saposhnikovia divaricata (Turcz. ex Ledeb.) Schischk. [Apiaceae; Saposhnikoviae Radix]; Angelica dahurica (Hoffm.) Benth. & Hook.f. ex Franch. & Sav. [Apiaceae; Angelicae Dahuricae Radix]; Glycyrrhiza uralensis Fisch. [Fabaceae; Glycyrrhizae Radix et Rhizoma]; and Ligusticum chuanxiong Hort. [Apiaceae; Chuanxiong Rhizoma]. Maize starch and magnesium stearate are listed as excipients. | The study identified Xiangju capsules as the intervention and reported the dosage regimen only. The full composition was not reported in the original article and was supplemented from product information. |
| Liang et al. | 2016 | Xiangju capsules | Huaxiangshu fruit infructescence with seeds removed, Platycarya strobilacea Siebold & Zucc. [Juglandaceae]; Prunella vulgaris L. [Lamiaceae; Prunellae Spica]; Chrysanthemum indicum L. [Asteraceae; Chrysanthemi Indici Flos]; Astragalus mongholicus Bunge [Fabaceae; Astragali Radix]; Magnolia biondii Pamp. [Magnoliaceae; Magnoliae Flos]; Saposhnikovia divaricata (Turcz. ex Ledeb.) Schischk. [Apiaceae; Saposhnikoviae Radix]; Angelica dahurica (Hoffm.) Benth. & Hook.f. ex Franch. & Sav. [Apiaceae; Angelicae Dahuricae Radix]; Glycyrrhiza uralensis Fisch. [Fabaceae; Glycyrrhizae Radix et Rhizoma]; and Ligusticum chuanxiong Hort. [Apiaceae; Chuanxiong Rhizoma]. Maize starch and magnesium stearate are listed as excipients. | The study identified Xiangju capsules as the intervention and reported the manufacturer, specification, and approval number. The full composition was not reported in the original article and was supplemented from product information. |
| Sun et al. | 2015 | Xiangju capsules | Huaxiangshu fruit infructescence with seeds removed, Platycarya strobilacea Siebold & Zucc. [Juglandaceae]; Prunella vulgaris L. [Lamiaceae; Prunellae Spica]; Chrysanthemum indicum L. [Asteraceae; Chrysanthemi Indici Flos]; Astragalus mongholicus Bunge [Fabaceae; Astragali Radix]; Magnolia biondii Pamp. [Magnoliaceae; Magnoliae Flos]; Saposhnikovia divaricata (Turcz. ex Ledeb.) Schischk. [Apiaceae; Saposhnikoviae Radix]; Angelica dahurica (Hoffm.) Benth. & Hook.f. ex Franch. & Sav. [Apiaceae; Angelicae Dahuricae Radix]; Glycyrrhiza uralensis Fisch. [Fabaceae; Glycyrrhizae Radix et Rhizoma]; and Ligusticum chuanxiong Hort. [Apiaceae; Chuanxiong Rhizoma]. Maize starch and magnesium stearate are listed as excipients. | The study identified Xiangju capsules as the intervention. The abstract image indicates combined treatment with budesonide and Xiangju capsules, but the full formulation details were not reported in the original article. The complete composition was supplemented from product information. |
| Cao et al. | 2014 | Xiangju capsules | Huaxiangshu fruit infructescence with seeds removed, Platycarya strobilacea Siebold & Zucc. [Juglandaceae]; Prunella vulgaris L. [Lamiaceae; Prunellae Spica]; Chrysanthemum indicum L. [Asteraceae; Chrysanthemi Indici Flos]; Astragalus mongholicus Bunge [Fabaceae; Astragali Radix]; Magnolia biondii Pamp. [Magnoliaceae; Magnoliae Flos]; Saposhnikovia divaricata (Turcz. ex Ledeb.) Schischk. [Apiaceae; Saposhnikoviae Radix]; Angelica dahurica (Hoffm.) Benth. & Hook.f. ex Franch. & Sav. [Apiaceae; Angelicae Dahuricae Radix]; Glycyrrhiza uralensis Fisch. [Fabaceae; Glycyrrhizae Radix et Rhizoma]; and Ligusticum chuanxiong Hort. [Apiaceae; Chuanxiong Rhizoma]. Maize starch and magnesium stearate are listed as excipients. | The study identified Xiangju capsules as the intervention, but did not report detailed formulation information. The full composition was supplemented from product information. |
| Shi et al. | 2012 | Xiangju capsules | Huaxiangshu fruit infructescence with seeds removed, Platycarya strobilacea Siebold & Zucc. [Juglandaceae]; Prunella vulgaris L. [Lamiaceae; Prunellae Spica]; Chrysanthemum indicum L. [Asteraceae; Chrysanthemi Indici Flos]; Astragalus mongholicus Bunge [Fabaceae; Astragali Radix]; Magnolia biondii Pamp. [Magnoliaceae; Magnoliae Flos]; Saposhnikovia divaricata (Turcz. ex Ledeb.) Schischk. [Apiaceae; Saposhnikoviae Radix]; Angelica dahurica (Hoffm.) Benth. & Hook.f. ex Franch. & Sav. [Apiaceae; Angelicae Dahuricae Radix]; Glycyrrhiza uralensis Fisch. [Fabaceae; Glycyrrhizae Radix et Rhizoma]; and Ligusticum chuanxiong Hort. [Apiaceae; Chuanxiong Rhizoma]. Maize starch and magnesium stearate are listed as excipients. | The study identified Xiangju capsules as the intervention and the discussion section described the nine main ingredients. However, the full product information was not reported in a standardized format. Excipients and the final complete formulation were supplemented from product information. |
| Shi et al. | 2010 | Xiangju capsules | Huaxiangshu fruit infructescence with seeds removed, Platycarya strobilacea Siebold & Zucc. [Juglandaceae]; Prunella vulgaris L. [Lamiaceae; Prunellae Spica]; Chrysanthemum indicum L. [Asteraceae; Chrysanthemi Indici Flos]; Astragalus mongholicus Bunge [Fabaceae; Astragali Radix]; Magnolia biondii Pamp. [Magnoliaceae; Magnoliae Flos]; Saposhnikovia divaricata (Turcz. ex Ledeb.) Schischk. [Apiaceae; Saposhnikoviae Radix]; Angelica dahurica (Hoffm.) Benth. & Hook.f. ex Franch. & Sav. [Apiaceae; Angelicae Dahuricae Radix]; Glycyrrhiza uralensis Fisch. [Fabaceae; Glycyrrhizae Radix et Rhizoma]; and Ligusticum chuanxiong Hort. [Apiaceae; Chuanxiong Rhizoma]. Maize starch and magnesium stearate are listed as excipients. | The study identified Xiangju capsules as the intervention and reported the specification and dosage. The full composition was not reported in the original article and was supplemented from product information. |

Supplementary Table 4. GRADE evidence quality assessment.

| **Certainty assessment** | | | | | | | **№ of patients** | | **Effect** | | **Certainty** | **Importance** |
| --- | --- | --- | --- | --- | --- | --- | --- | --- | --- | --- | --- | --- |
| **№ of studies** | **Study design** | **Risk of bias** | **Inconsistency** | **Indirectness** | **Imprecision** | **Other considerations** | **[干预]** | **[对照]** | **Relative (95% CI)** | **Absolute (95% CI)** |  |  |
| **Overall effective rate** | | | | | | | | | | | | |
| 13 | randomised trials | serious | not serious | not serious | not serious | publication bias strongly suspected | 747/793 (94.2%) | 662/790 (83.8%) | **RR 1.12** (1.08 to 1.16) | **101 more per 1,000** (from 67 more to 134 more) | ⨁⨁◯◯ Low |  |
| **IL-4** | | | | | | | | | | | | |
| 8 | randomised trials | serious | very serious | not serious | not serious | none | 411 | 411 | - | SMD **1.55 SD lower** (1.93 lower to 1.16 lower) | ⨁◯◯◯ Very low |  |
| **IL-12** | | | | | | | | | | | | |
| 4 | randomised trials | serious | very serious | not serious | not serious | none | 227 | 227 | - | SMD **1.76 SD higher** (0.79 higher to 2.73 higher) | ⨁◯◯◯ Very low |  |
| **IgE** | | | | | | | | | | | | |
| 6 | randomised trials | serious | very serious | not serious | not serious | none | 323 | 323 | - | SMD **1.55 SD lower** (2.92 lower to 0.18 lower) | ⨁◯◯◯ Very low |  |
| **Runny Nose Symptom Score** | | | | | | | | | | | | |
| 4 | randomised trials | serious | very serious | not serious | not serious | none | 255 | 255 | - | SMD **1.81 SD lower** (3.36 lower to 0.27 lower) | ⨁◯◯◯ Very low |  |
| **Sneezing Symptom Score** | | | | | | | | | | | | |
| 4 | randomised trials | serious | very serious | not serious | not serious | none | 255 | 255 | - | SMD **1.14 SD lower** (1.86 lower to 0.41 lower) | ⨁◯◯◯ Very low |  |
| **Nasal Congestion Symptom Score** | | | | | | | | | | | | |
| 3 | randomised trials | serious | very serious | not serious | not serious | none | 219 | 219 | - | SMD **0.15 SD higher** (1.41 lower to 1.72 higher) | ⨁◯◯◯ Very low |  |
| **Adverse Events** | | | | | | | | | | | | |
| 7 | randomised trials | serious | not serious | not serious | not serious | none | 23/407 (5.7%) | 32/407 (7.9%) | **RR 0.72** (0.43 to 1.20) | **22 fewer per 1,000** (from 45 fewer to 16 more) | ⨁⨁⨁◯ Moderate |  |

Supplementary Figure 1. Sensitivity analysis of overall effective rate


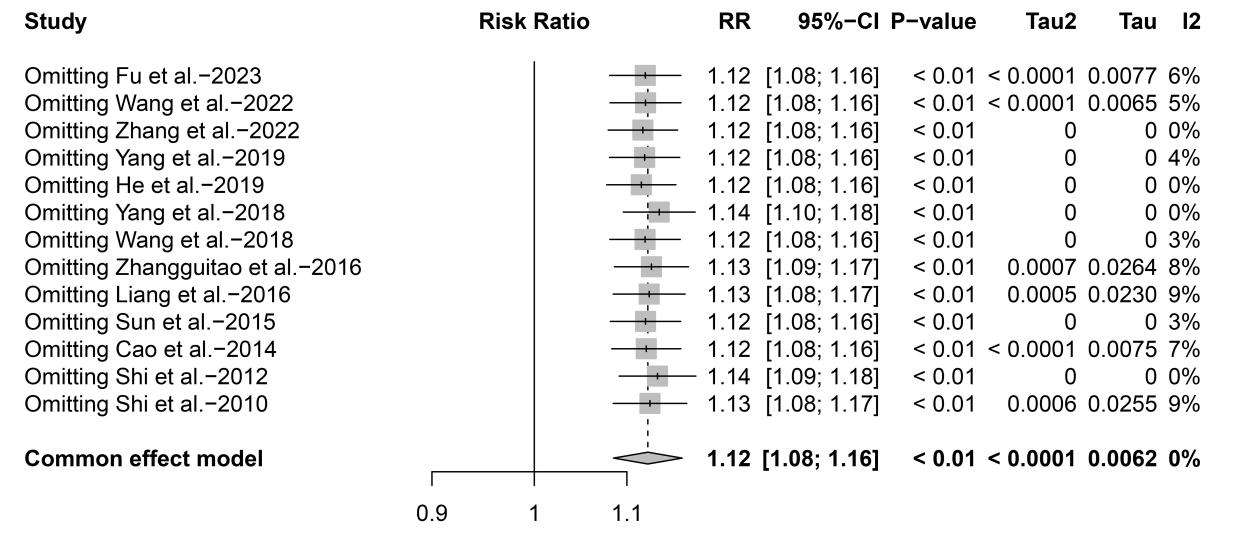


Supplementary Figure 2. Sensitivity analysis of IL-4 levels


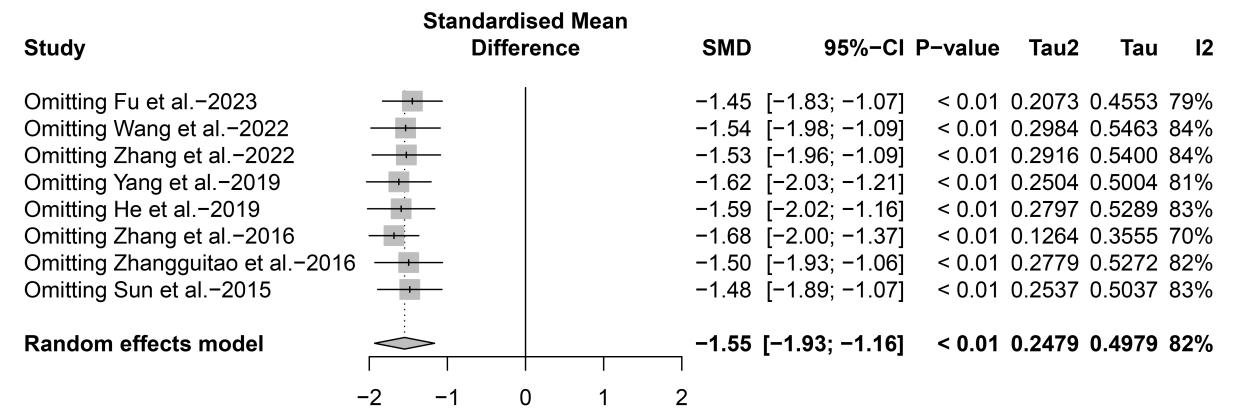


Supplementary Figure 3. Sensitivity analysis of IL-12 levels


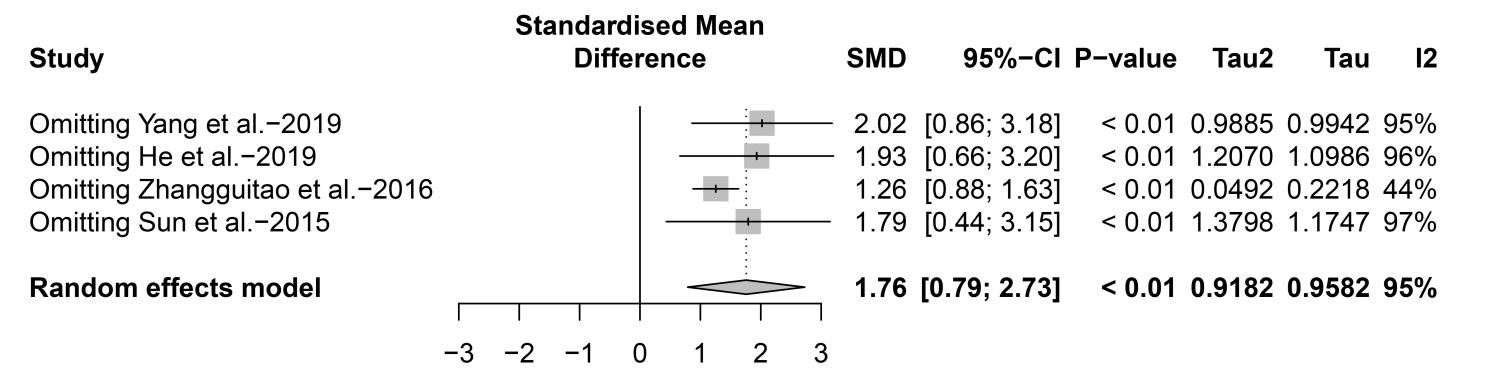


Supplementary Figure 4. Sensitivity analysis of IgE levels


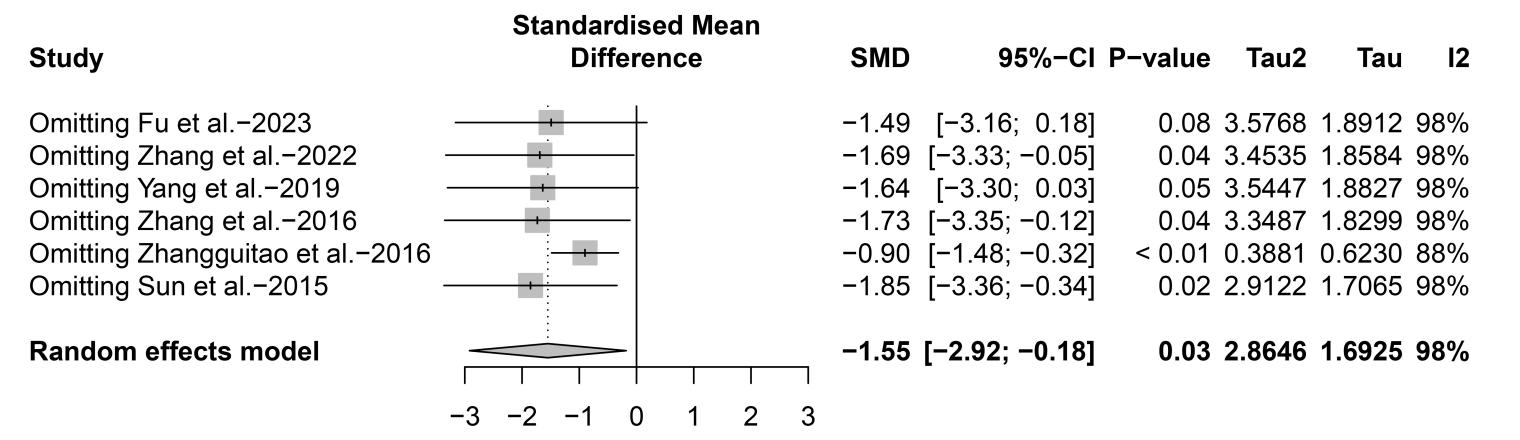


Supplementary Figure 5. Sensitivity analysis of runny nose symptom scores


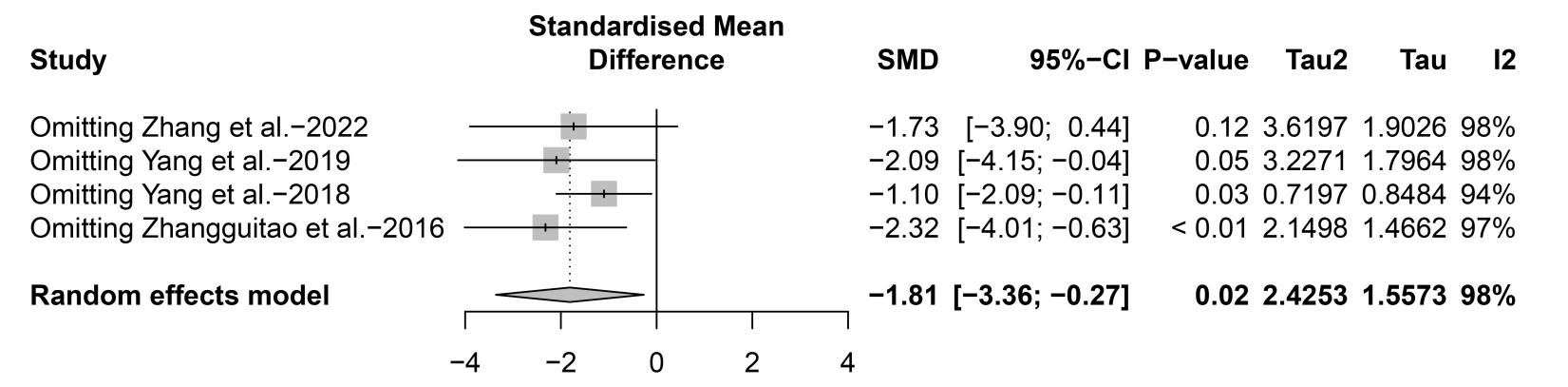


Supplementary Figure 6. Sensitivity analysis of sneezing symptom scores


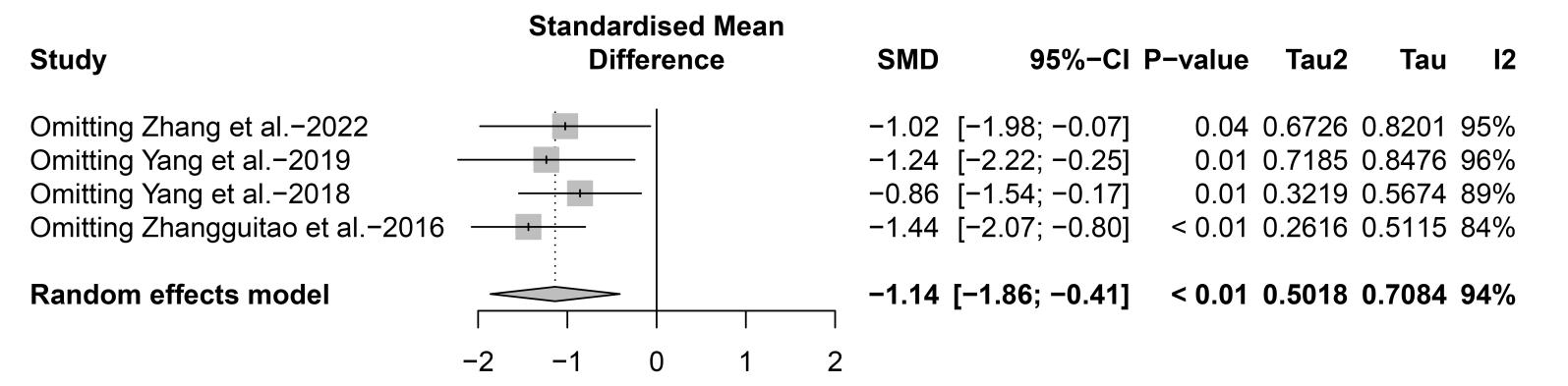


Supplementary Figure 7. Sensitivity analysis of nasal congestion symptom scores


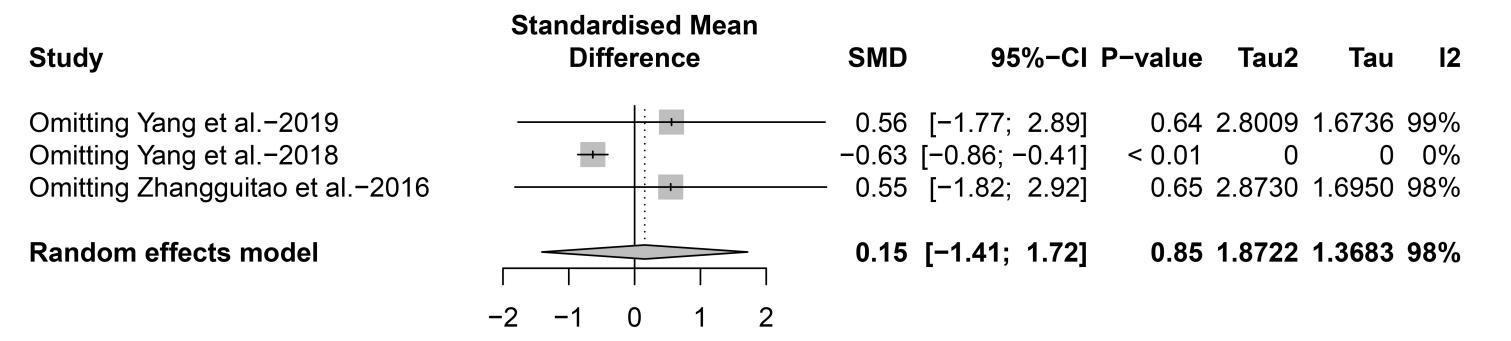


Supplementary Figure 8. Sensitivity analysis of adverse events


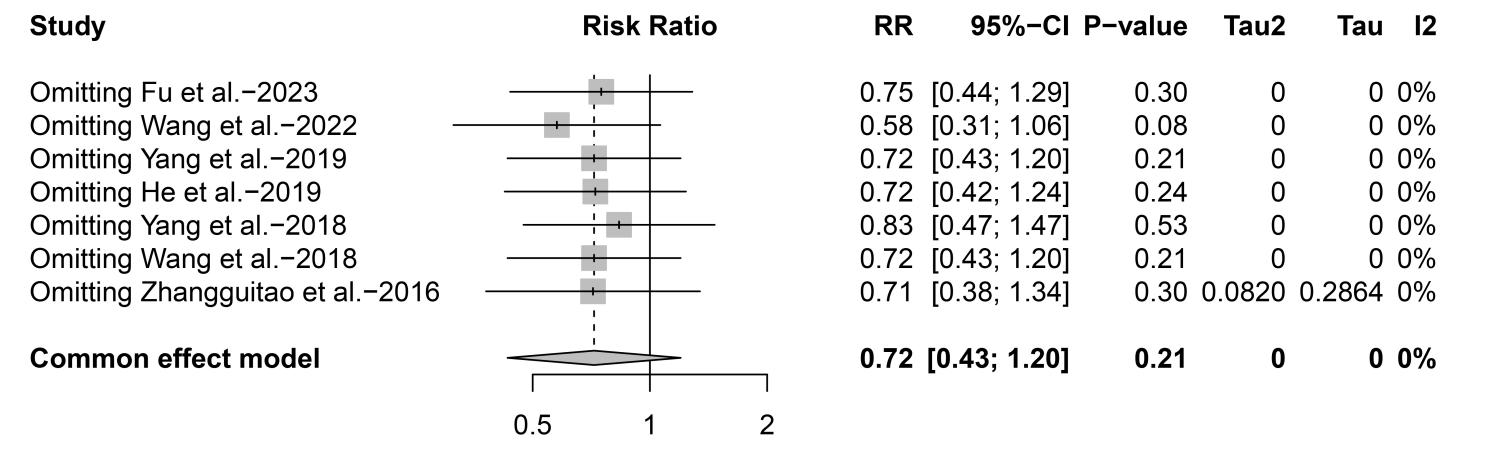


Supplementary Figure 9. Subgroup analysis of overall effective rate by age


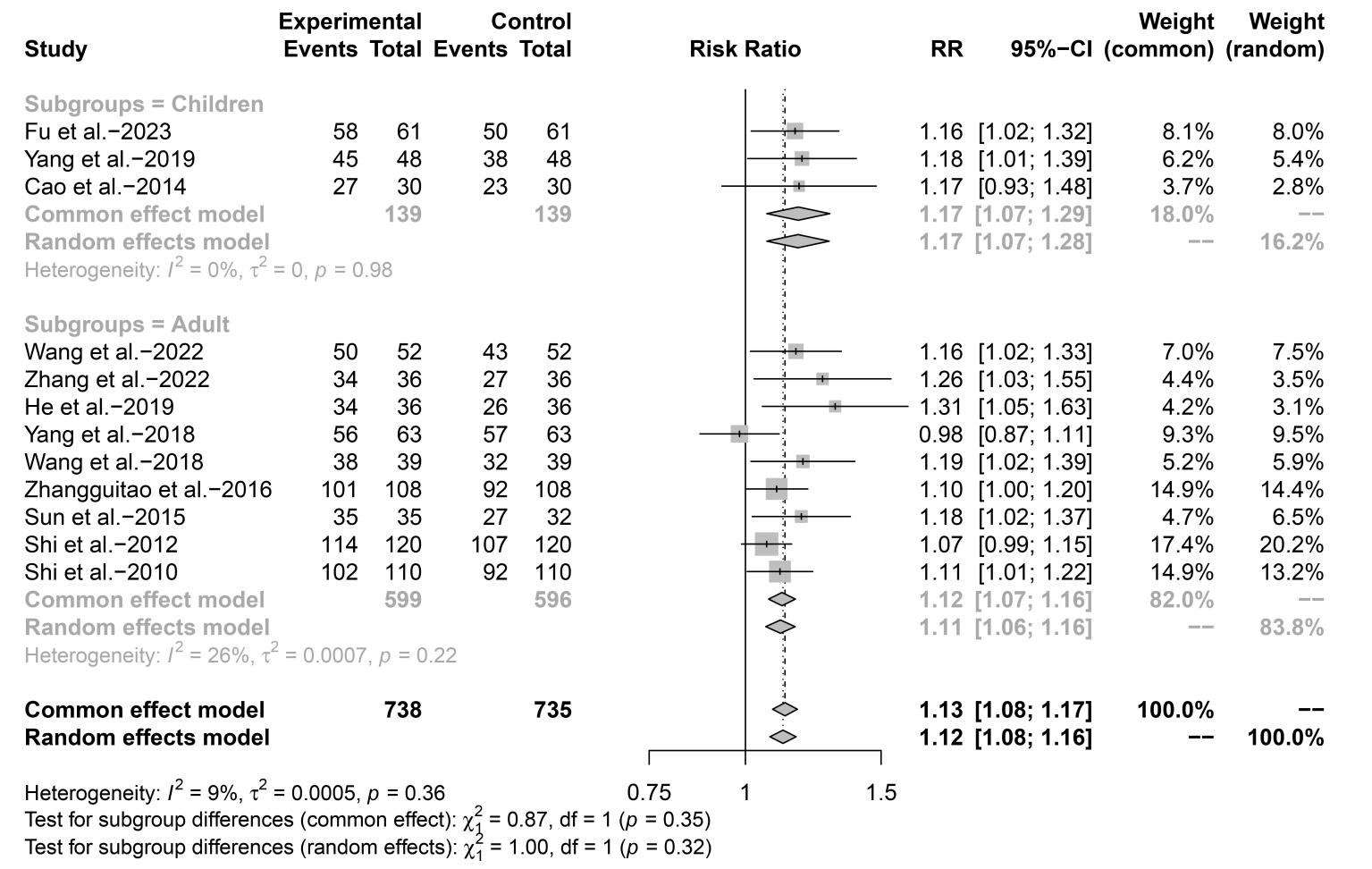


Supplementary Figure 10. Subgroup analysis of IL-4 levels by age


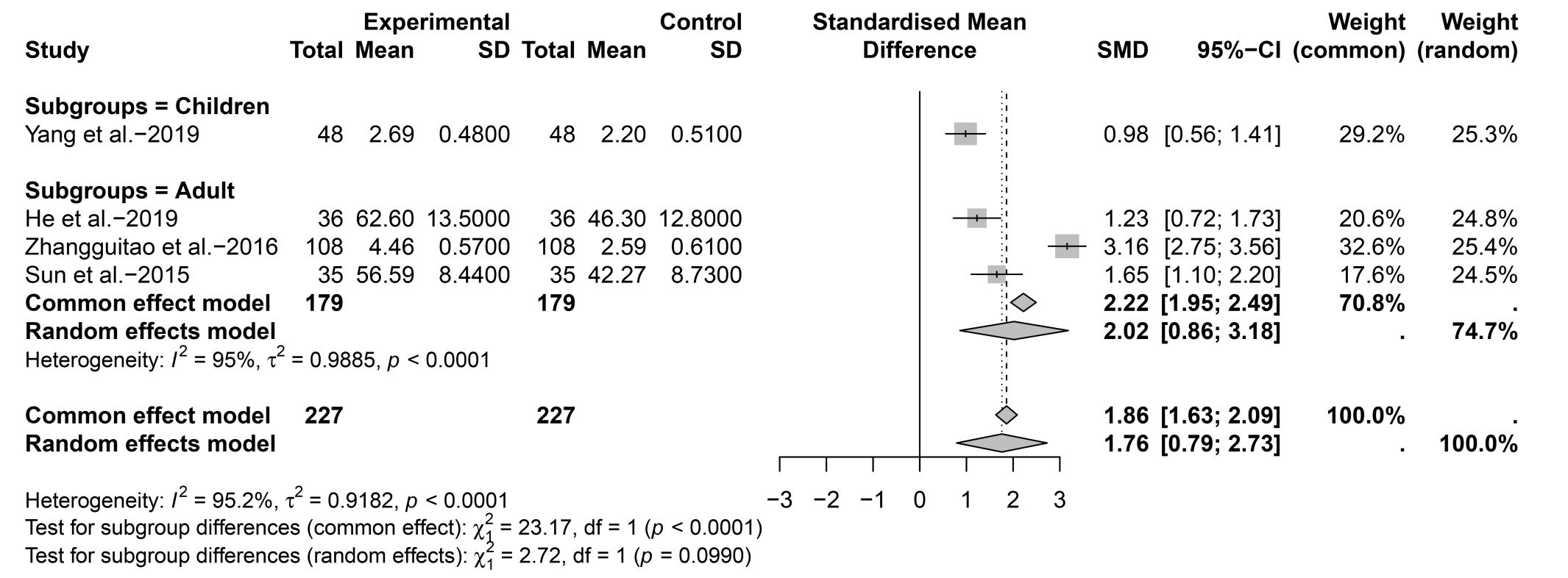


Supplementary Figure 11. Subgroup analysis of IL-12 levels by age


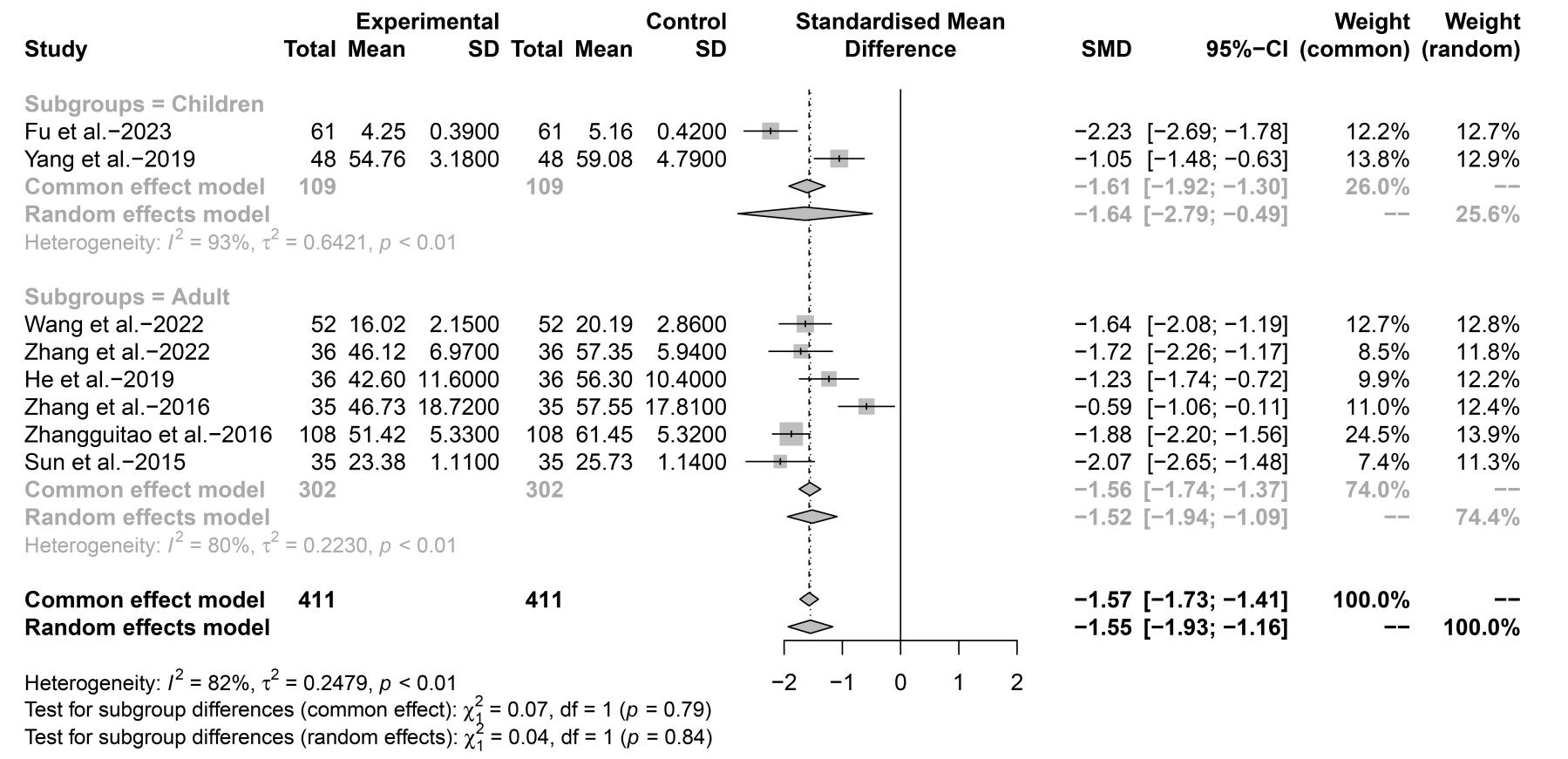


Supplementary Figure 12. Subgroup analysis of IgE levels by age


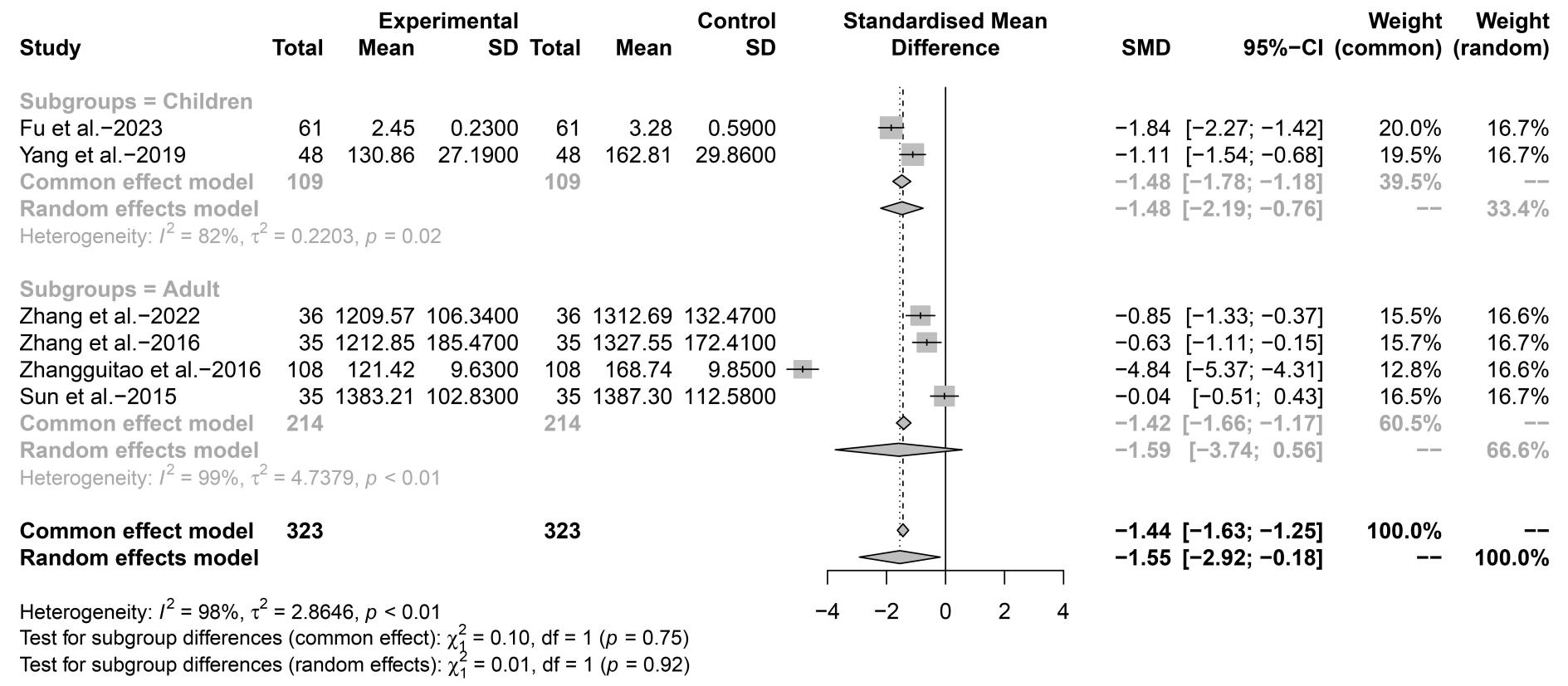


Supplementary Figure 13. Subgroup analysis of runny nose scores by age


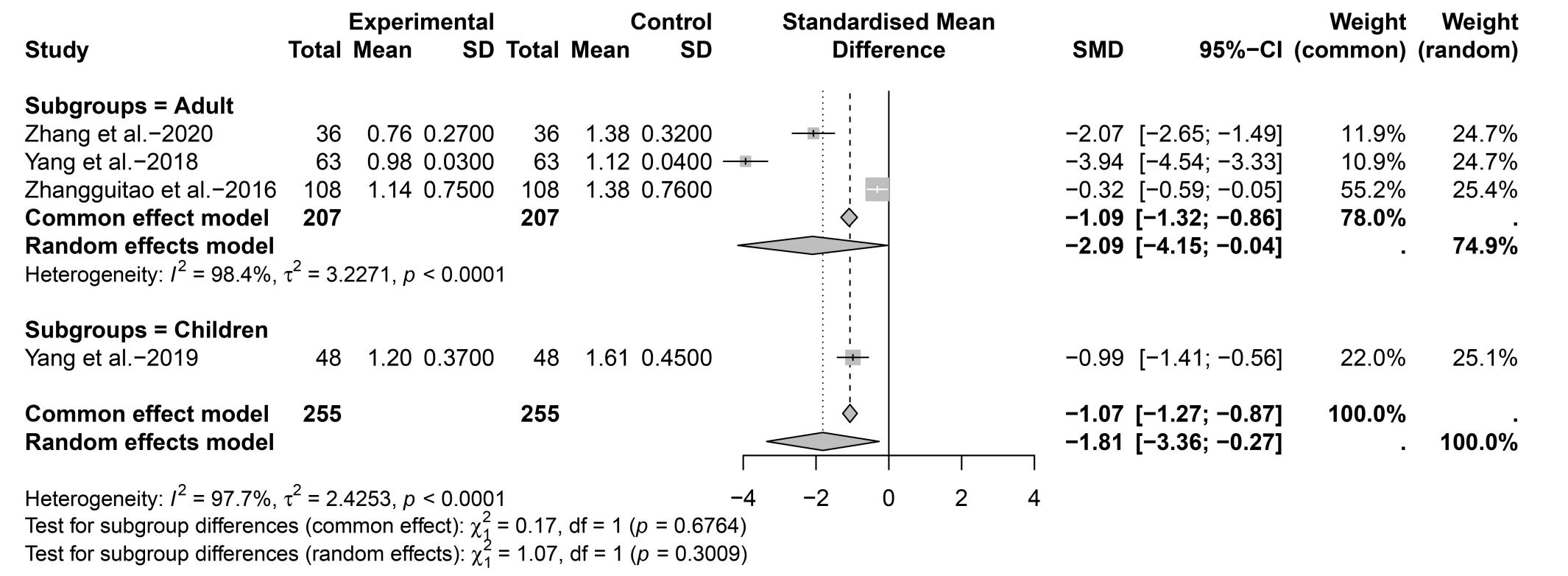


Supplementary Figure 14. Subgroup analysis of sneezing symptom score by age


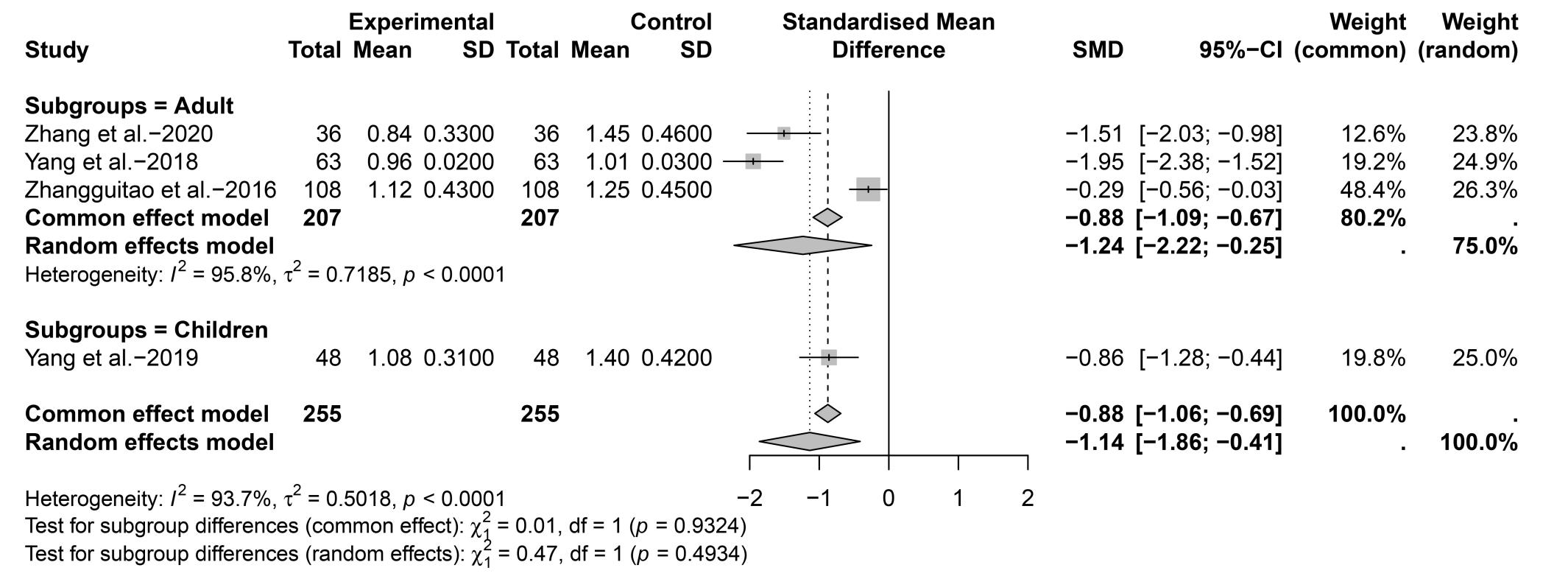


Supplementary Figure 15. Subgroup analysis of nasal congestion symptom score by age


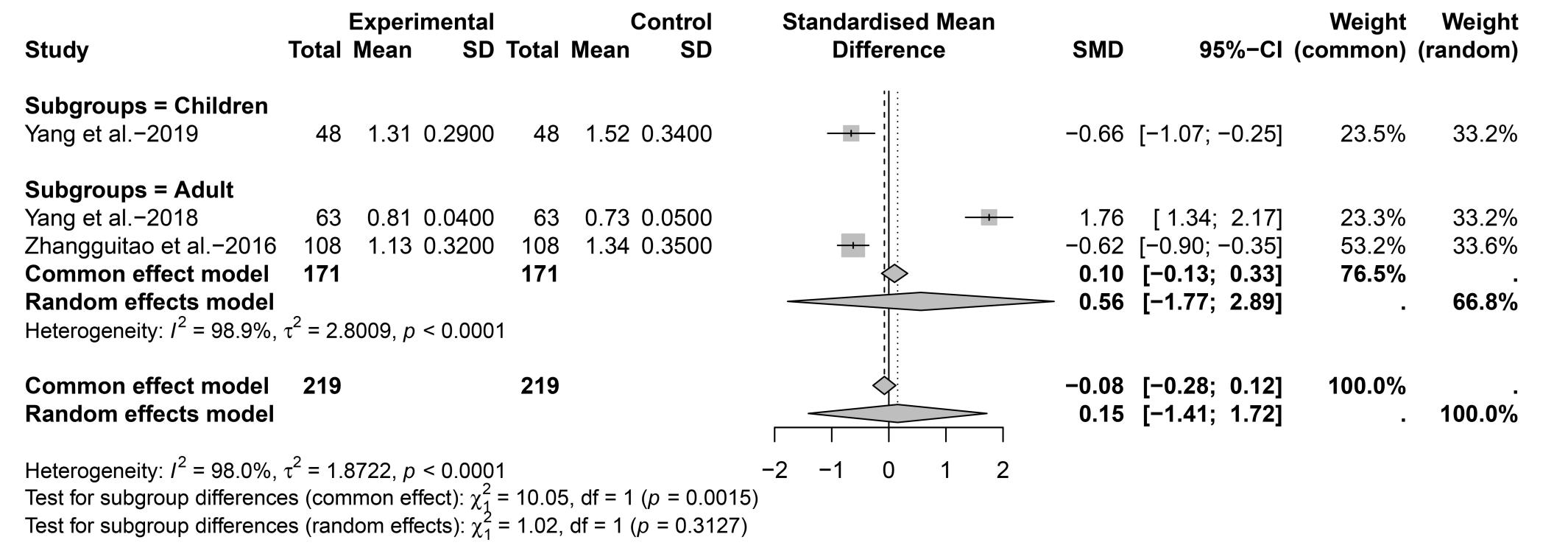


Supplementary Figure 16. Subgroup analysis of adverse events by age


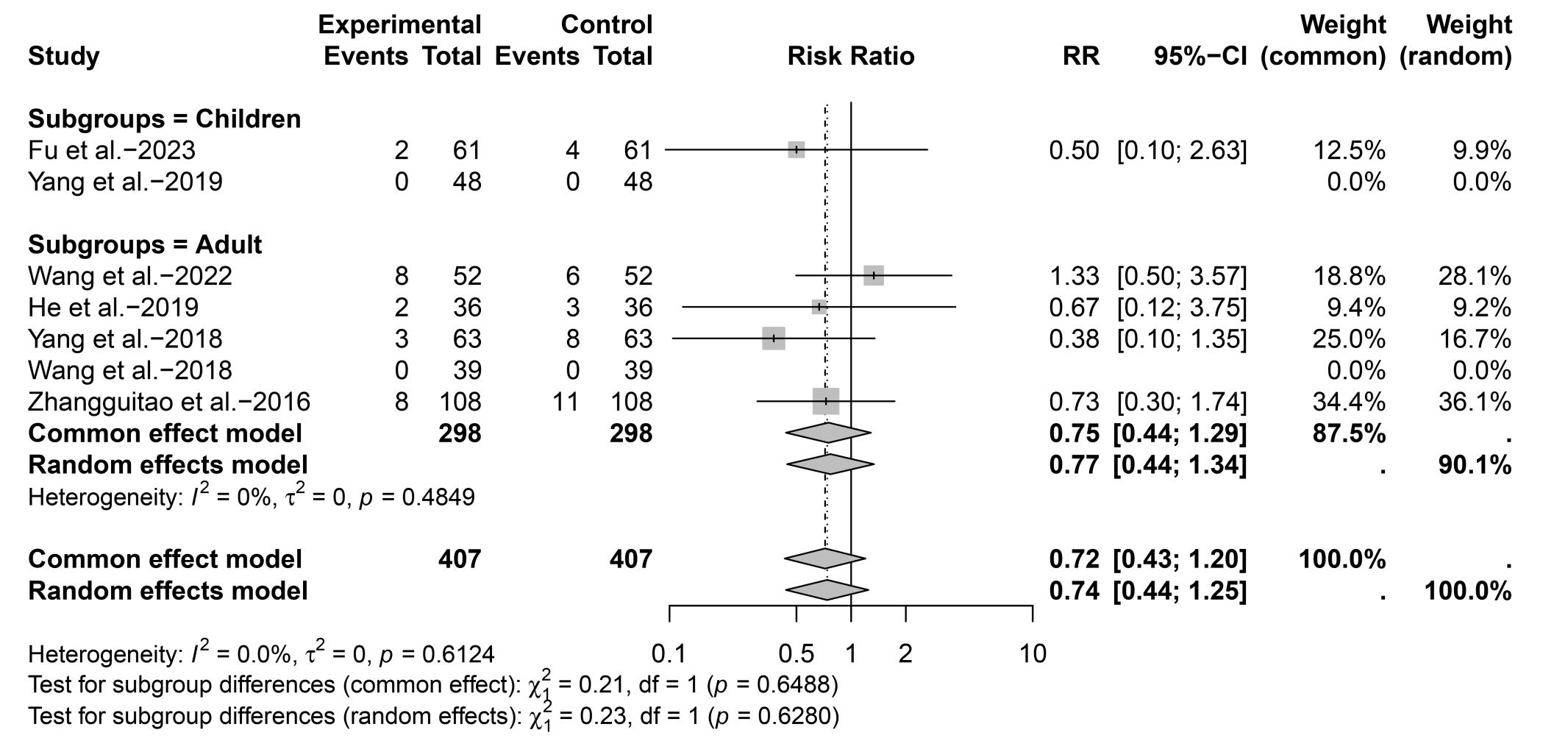


Supplementary Figure 17. Subgroup analysis of overall effective rate by combination regimen


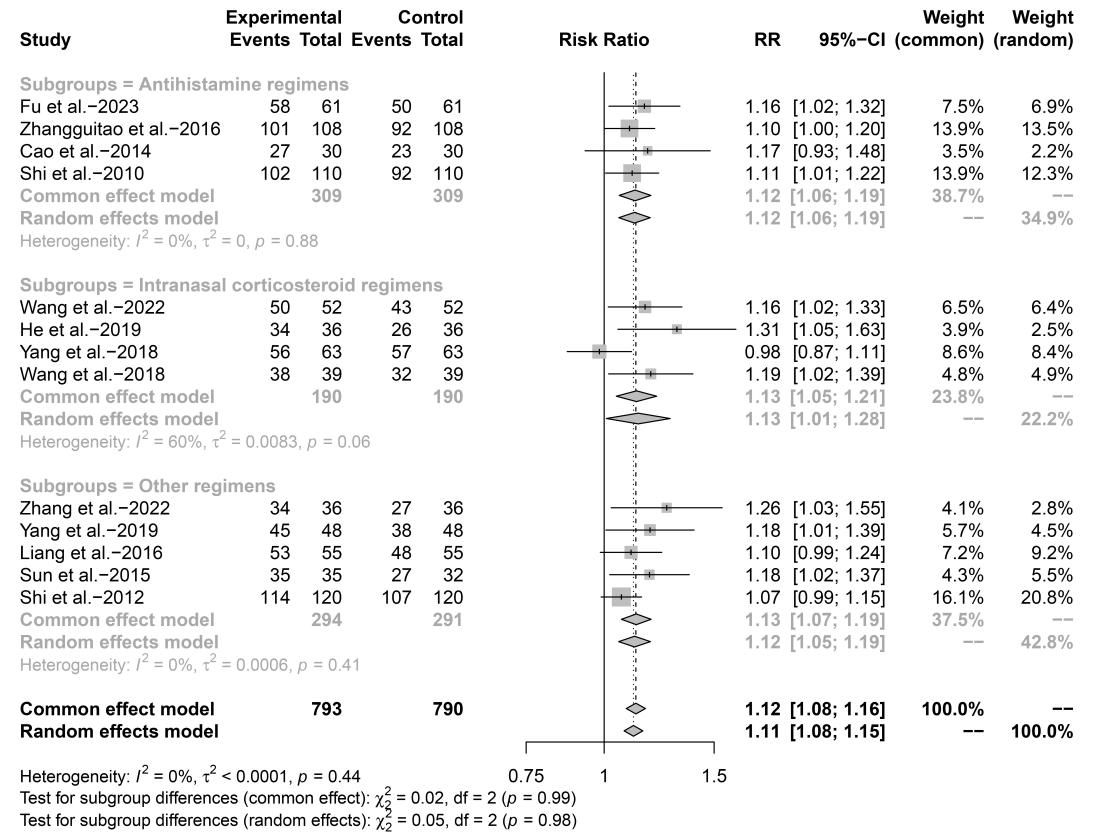


Supplementary Figure 18. Subgroup analysis of IL-4 levels by combination regimen


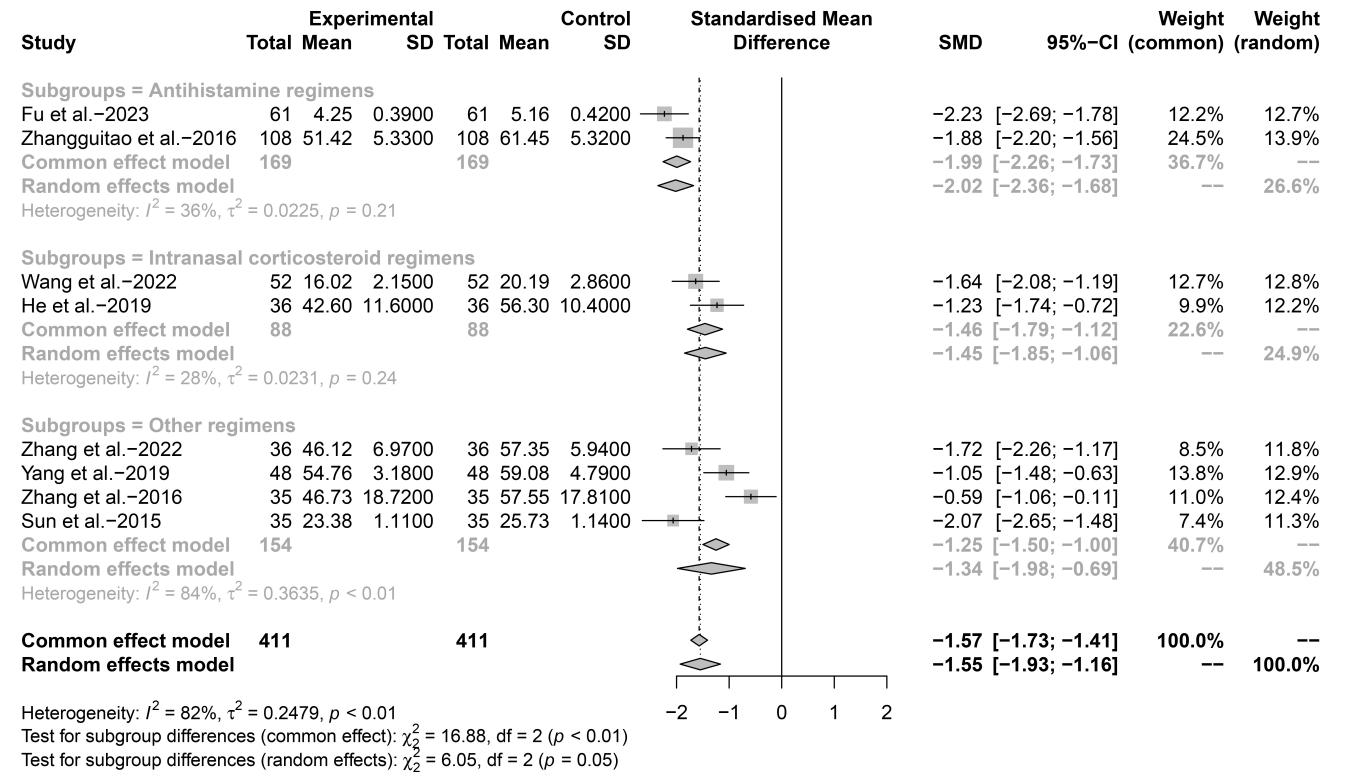


Supplementary Figure 19. Subgroup analysis of IgE levels by combination regimen


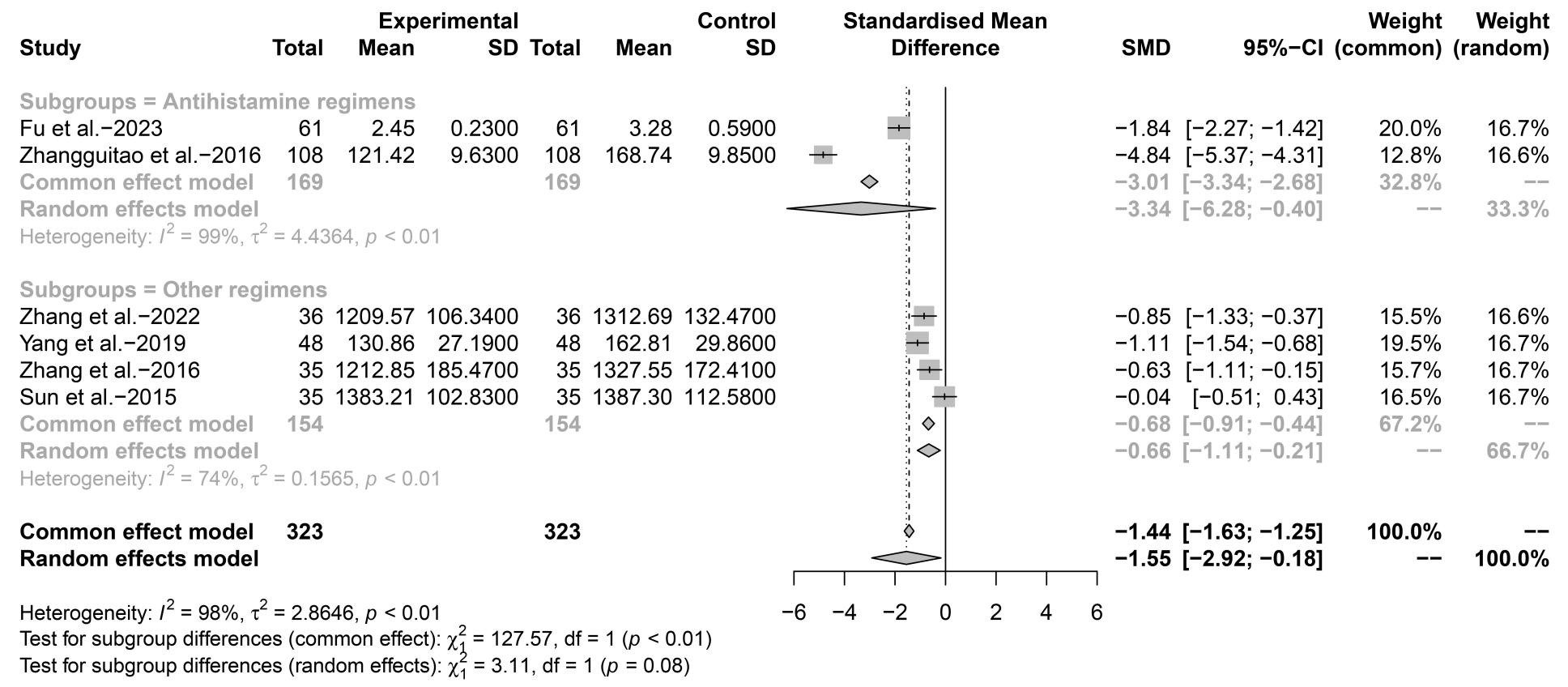


Supplementary Figure 20. Subgroup analysis of overall effective rate by treatment duration


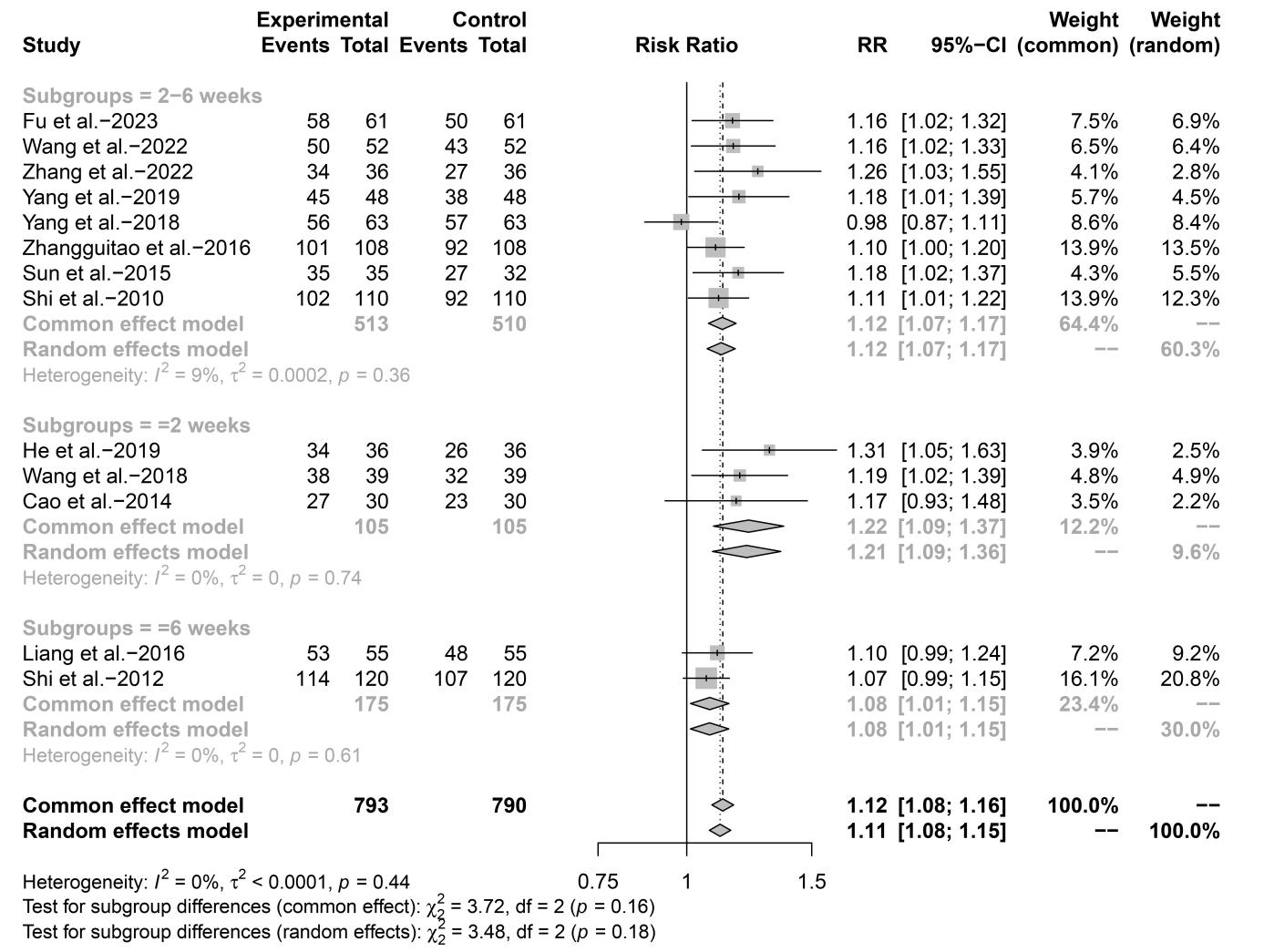


Supplementary Figure 21. Subgroup analysis of IL-4 levels by treatment duration


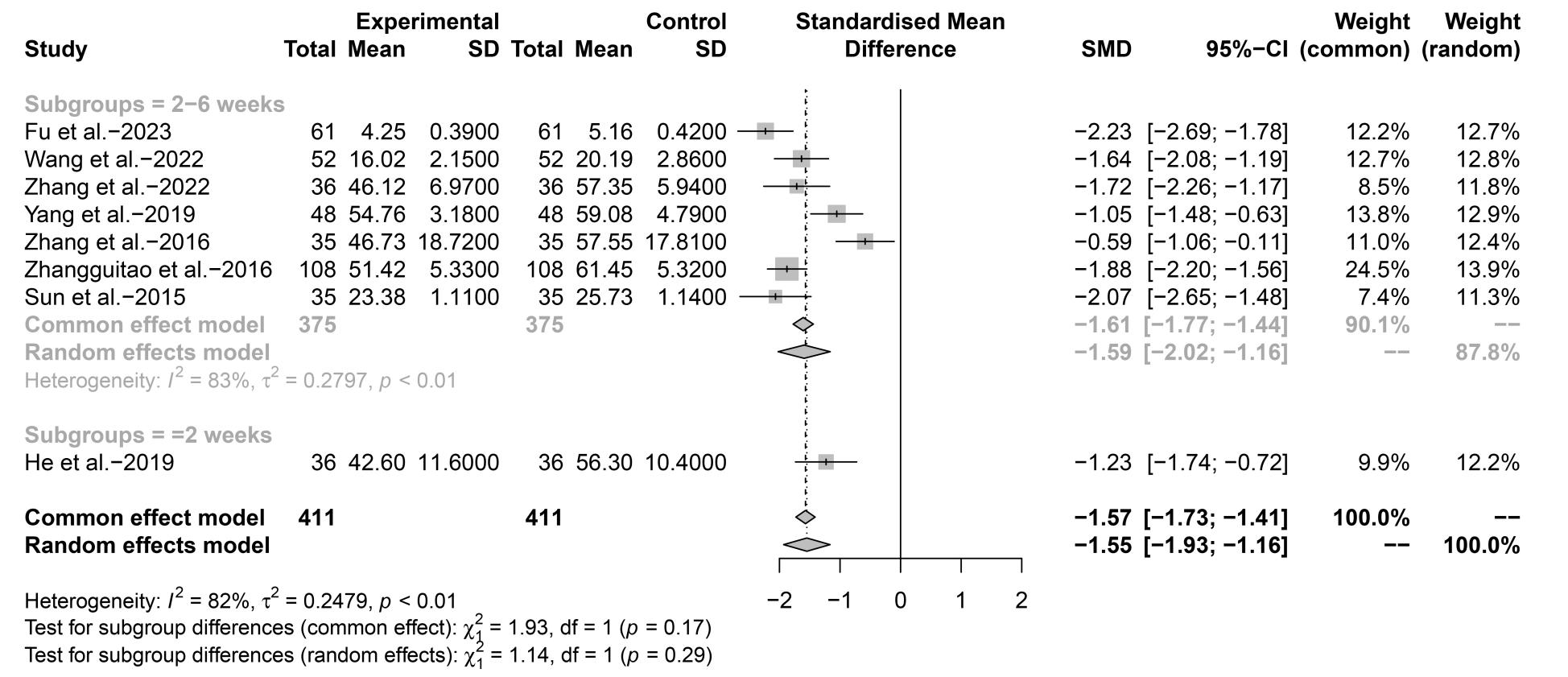


Supplementary Figure 22. Trim-and-fill analysis for overall effective rate


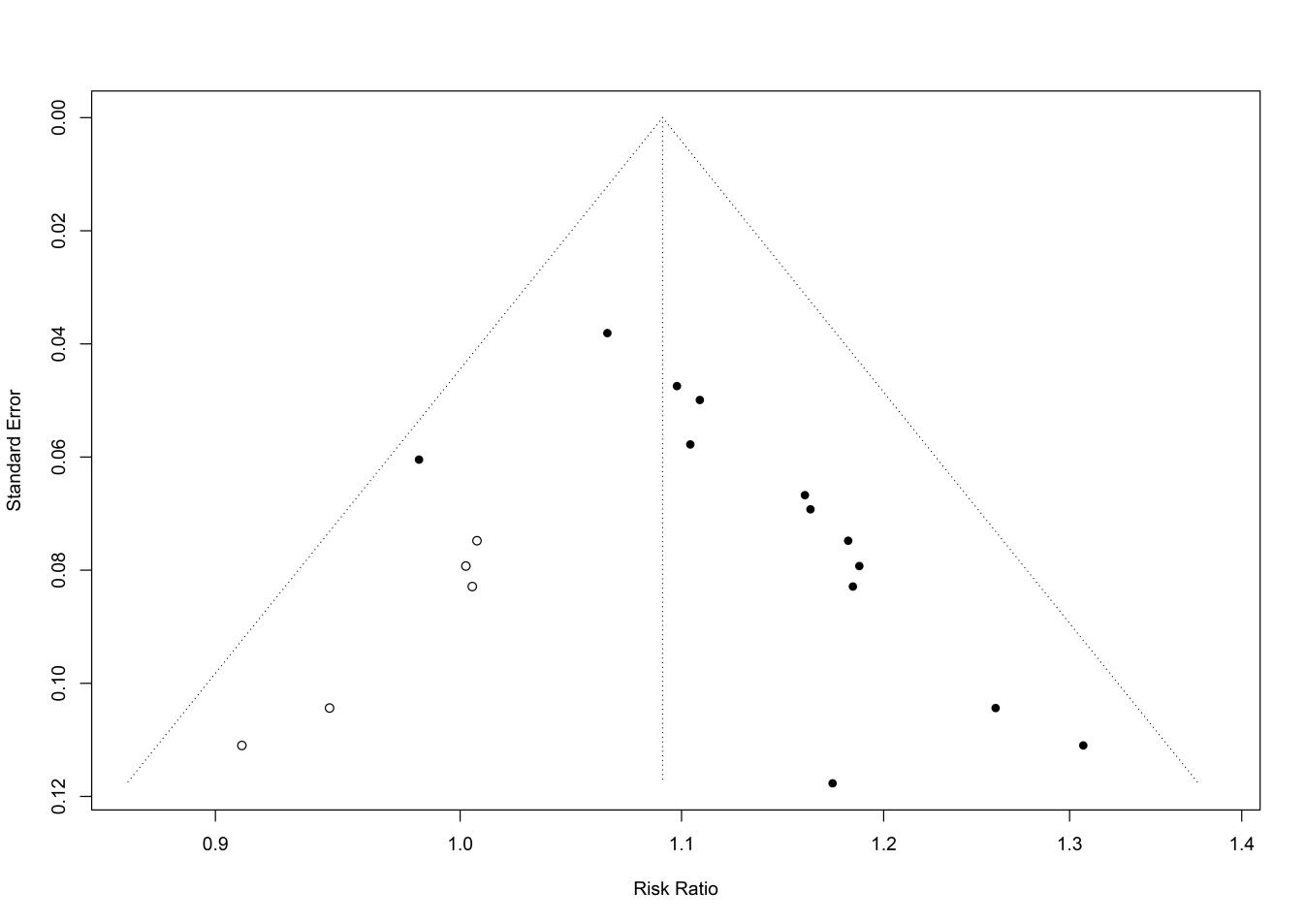

Supplement: Supplementary file 1 [file DataSheet1.docx]
